# Exploring diversity rock-inhabiting fungi from northern Thailand: a new genus and three new species belonged to the family *Herpotrichiellaceae*

**DOI:** 10.3389/fcimb.2023.1252482

**Published:** 2023-08-25

**Authors:** Tanapol Thitla, Jaturong Kumla, Sinang Hongsanan, Chanokned Senwanna, Surapong Khuna, Saisamorn Lumyong, Nakarin Suwannarach

**Affiliations:** ^1^ Master of Science Program in Applied Microbiology (International Program), Faculty of Science, Chiang Mai University, Chiang Mai, Thailand; ^2^ Department of Biology, Faculty of Science, Chiang Mai University, Chiang Mai, Thailand; ^3^ Center of Excellence in Microbial Diversity and Sustainable Utilization, Chiang Mai University, Chiang Mai, Thailand; ^4^ Academy of Science, The Royal Society of Thailand, Bangkok, Thailand

**Keywords:** herpotrichiellaceous fungi, new taxa, rock-inhabiting fungi, taxonomy, tropical area

## Abstract

Members of the family *Herpotrichiellaceae* are distributed worldwide and can be found in various habitats including on insects, plants, rocks, and in the soil. They are also known to be opportunistic human pathogens. In this study, 12 strains of rock-inhabiting fungi that belong to *Herpotrichiellaceae* were isolated from rock samples collected from forests located in Lamphun and Sukhothai provinces of northern Thailand during the period from 2021 to 2022. On the basis of the morphological characteristics, growth temperature, and multi-gene phylogenetic analyses of a combination of the internal transcribed spacer, the large subunit, and the small subunit of ribosomal RNA, beta tubulin and the translation elongation factor 1-a genes, the new genus, *Petriomyces* gen. nov., has been established to accommodate the single species, *Pe. obovoidisporus* sp. nov. In addition, three new species of *Cladophialophora* have also been introduced, namely, *Cl. rupestricola*, *Cl. sribuabanensis*, and *Cl. thailandensis*. Descriptions, illustrations, and a phylogenetic trees indicating the placement of these new taxa are provided. Here, we provide updates and discussions on the phylogenetic placement of other fungal genera within *Herpotrichiellaceae*.

## Introduction

*Herpotrichiellaceae* is a family within order *Chaetothyriales* (class *Eurotiomycetes*), which was introduced by Munk (1953) to accommodate *Herpotrichiella*, with *H. moravica* as the type species. Accordingly, *Herpotrichiella moravica* is now considered a synonym of *Capronia moravica* based on the teleomorph–anamorph connection of *Herpotrichiellaceae* (Müller et al., 1987). Likewise, the morphology of *Capronia moravica* and *Ca. pilosella* has been found to be identical; thus, *Ca. moravica* is now synonymized under *Ca. pilosella* (Untereiner, 1997). *Capronia* is the older name that was introduced by Saccardo (1883); therefore, *Capronia* has been retained over *Herpotrichiella* (Untereiner, 1997; Quan et al., 2020). *Herpotrichiellaceae* is characterized by having setose, ostiolate ascomata, and bitunicate asci with endotunica; by appearing greenish gray to brown; and by having didymosporous, phragmosporous, or dictyosporous ascospores (Müller et al., 1987; Untereiner et al., 1995; Untereiner, 1997; Tian et al., 2021). The asexual morphs of the family are characterized as dematiaceous hyphomycetes, and commonly referred to as black yeast (Müller et al., 1987; Untereiner et al., 1995; Tian et al., 2021). This family is well-known to consist of a sexual morph as a genus *Capronia* and several asexual morph genera, such as *Cladophialophora*, *Exophiala*, *Fonsecaea*, *Phialophora*, and *Rhinocladiella* (de Hoog et al., 2011; Phukhamsakda et al., 2022). Due to the similar morphological features of these asexual genera, the morphological identification of these asexual genera can be challenging (Badali et al., 2008; Yang et al., 2021). Therefore, a combination of morphological and molecular data is needed to identify the species in this family and to clarify their phylogenetic placement. Twenty genera are currently accepted in *Herpotrichiellaceae*; namely, *Aculeata*, *Atrokylindriopsis*, *Brycekendrickomyces*, *Capronia*, *Cladophialophora*, *Exophiala*, *Fonsecaea*, *Marinophialophora*, *Melanoctona*, *Metulocladosporiella*, *Minimelanolocus*, *Neosorocybe*, *Phialophora*, *Pleomelogramma*, *Rhinocladiella*, *Sorocybe*, *Thysanorea*, *Uncispora*, *Valentiella*, and *Veronaea* (Wijayawardene et al., 2020; Tian et al., 2021; Bezerra et al., 2022; Wijayawardene et al., 2022). The members of *Herpotrichiellaceae* have been collected from several sources worldwide. These consist of air, insects, lichens, plants, rocks, and soil (Diederich et al., 2013; Feng et al., 2014; Das et al., 2019; Libert et al., 2019; Lima et al., 2020; Sun et al., 2020; Thitla et al., 2022). In addition, numerous studies have reported that some herpotrichiellaceous fungi are opportunistic fungi in humans and other animals (Badali et al., 2008; de Hoog et al., 2011; Li et al., 2017). Several herpotrichiellaceous fungi were reported to survive under extreme conditions (high and low temperatures, desiccation, lack of nutrients, and UV radiation), such as the species within genera *Exophiala* and *Cladophialophora* (Seyedmousavi et al., 2011; Gostinčar et al., 2022; Tesei, 2022). The accumulation of melanin in the cell walls of these fungi is one of the key factors to survival in extreme conditions (de Hoog et al., 2011; Li et al., 2017; Coleine and Selbmann, 2021).

*Cladophialophora* is one of the genera in the family *Herpotrichiellaceae* and has been reported in many regions of the world including Africa, Asia, Europe, North America, Oceania, and South America (de Hoog et al., 1995; de Hoog et al., 2007; Badali et al., 2008; Badali et al., 2011; Madrid et al., 2016; Sun et al., 2020; Nascimento et al., 2021). The genus was introduced by Borelli (1980) with *Cl*. *ajelloi* Borelli as the type species [currently named *Cl*. *carrionii* (Badali et al., 2008; Boonmee et al., 2021). Presently, there are 53 records of *Cladophialophora* in the Index Fungorum database (Index Fungorum, 2023). Of those, some species may belong to different genera. For example, *Cl*. *brevicatenata*, *Cl*. *hachijoensis*, and *Cl*. *kellermaniana* were subsequently transferred to *Tyrannosorus hanlinianus*, *Pseudocladosporium hachijoense*, and *Chalastospora gossypii*, respectively (Braun, 1998; Crous et al., 2009; Shen et al., 2020). However, 49 *Cladophialophora* species have currently been validated and accepted.

Rocks are one of the interesting natural habitats of fungi. Rock-inhabiting fungi were divided into two groups on the basis of their ecology and taxonomy. The first group comprises hyphomycetes of soil and epiphytic fungi (such as *Aureobasidium* or *Phoma*), whereas the second group includes melanized cell-walled fungi that exhibit slow growth, meristematic growth, or the production of yeast-like cells, typically belonging to the orders *Capnodoales*, *Chaetothyriales*, and *Dothideales* (Wollenzien et al., 1995; Gorbushina, 2007; Coleine and Selbmann, 2021). There are numerous reports on the biotechnological capabilities of rock-inhabiting fungi in astrobiology, radioprotection, biomedical, and bioremediation fields (Aureli et al., 2020; Lin and Xu, 2020; Tran-Ly et al., 2020; Coleine and Selbmann, 2021; Mattoon et al., 2021; Cassaro et al., 2022; Liu et al., 2022). Several species of herpotrichiellaceous fungi were previously isolated from rocks, such as *Cladophialophora nyingchiensis*, *Cl. tumulicola*, *Exophiala bonariae*, *Ex. clavispora*, and *Ex. siamensis* (Isola et al., 2016; Kiyuna et al., 2018; Sun et al., 2020; Thitla et al., 2022). In addition, numerous prior reports have highlighted Thailand as a hot spot for discovering new fungal species (Hyde et al., 2018; Khuna et al., 2022). Nonetheless, information on the rock-inhabiting fungi in Thailand is still limited. Thus, the main objective of this study is to study the diversity of rock-inhabiting fungi in Thailand. During our investigation, we identified 12 herpotrichiellaceous fungi, *viz*., 10 strains of *Cladophialophora* and two strains of unrecognized fungal taxa. Morphology, growth temperature, and multi-gene phylogenetic analyses indicate that four herpotrichiellaceous fungi are novel in *Herpotrichiellaceae*. In addition, we have updated the existing reference data on the members of *Herpotrichiellaceae*.

## Materials and methods

### Sample collection and fungal isolation

Rock samples appearing black fungal mycelia were collected from a dipterocarp forests in Lamphun (18°32′13″N 99°07′31″E, elevation at 432 m; and 18°32′11″N 99°07′22″E, elevation at 444 m) and Sukhothai (17°32′58″N 99°29′49″E, elevation at 153 m) provinces, Thailand, in 2021−2022. During the collection period, Lamphun province had daily rainfall of 6.5 mm, whereas Sukhothai province received daily rainfall of 2.3 mm. Temperatures in Lamphun province ranged from 22°C to 36°C, whereas temperatures in Sukhothai province ranged from 24°C to 38°C. Rock samples were obtained following the method described by Thitla et al. (2022). Fungi were isolated using an adaptation of the technique reported by Selbmann et al. (2014). Rock samples were cleaned in 1% sodium hypochlorite for 10 min before being rinsed five times with sterile and deionized water to eliminate any trace of detergent. To obtain the fungal strain, pounding rock samples and seeding rock shards were sprinkled onto malt extract agar (MEA; Gibco, Life Technologies Corporation, USA) and dichloran-rose bengal agar (DRBC; Difco, Becton, Dickinson and Company, USA) supplemented with chloramphenicol 100 mg/L. Plates were incubated at 25°C for 4 weeks, with daily inspections. Dark mycelia fungi were aseptically transferred to a MEA plates. Pure fungal strains were kept in 15% glycerol and deposited in the Culture Collection of Sustainable Development of Biological Resources Laboratory (SDBR), Faculty of Science, Chiang Mai University, Chiang Mai, Thailand. In addition, new fungal taxa were registered in the MycoBank database (MycoBank, 2023).

### Morphology and growth temperature

Macro-morphologies, including colony characterization, pigment production, and colony diameter, were investigated on potato dextrose agar (PDA; Condalab, Laboratorios Conda S.A., Spain), MEA, and oatmeal agar (OA; Difco, Becton, Dickinson and Company, USA) at 25°C in the dark for 28 days. To examine the fungal growth at different temperatures, the colony diameter was measured on MEA at 4°C, 10°C, 15°C, 20°C, 25°C, 28°C, 30°C, 35°C, 37°C, and 40°C for 4 weeks in darkness. Three replicates were performed for each fungal strain at each temperature. Micromorphological features were investigated using a light microscope (Nikon Eclipse Ni-U, Japan). The Tarosoft (R) Image was used to calculate the size of fungal structures (e.g., hyphae, conidiophore, conidiogenous cell, conidia, and chlamydospore).

### DNA extraction, amplification, and sequencing

Fungal genomic DNA of each fungal strain was extracted from mycelia grown on MEA at 25°C for 3 weeks, using s DNA extraction kit (FAVORGEN, Ping-Tung, Taiwan). The internal transcribed spacer (ITS), large subunit (nrLSU), and small subunit (nrSSU) of ribosomal RNA, beta tubulin gene (*tub2*), and the translation elongation factor 1-α (*tef1-α*) genes were amplified by polymerase chain reaction (PCR) using ITS5/ITS4 (White et al., 1990), LROR/LR5 (Vilgalys and Hester, 1990; Rehner and Samuels, 1994), NS1/NS4 (White et al., 1990), Bt2a/Bt2b (Glass and Donaldson, 1995), and EF1-728F/EF1-986R (Carbone and Kohn, 1999) primers, respectively. PCR amplifications were performed using 20 µL of reaction mixtures, consisting of 1 µL of genomic DNA, 1 µL of each primer, 10 µL of Quick TaqTM HS Dye-Mix (TOYOBO, Japan), and 7 µL of deionized water. The conditions of PCR reactions consisted of a first denaturation step performed at 95°C for 5 min and denaturation step at 95°C for 30 s; then, to amplify the ITS region, an annealing step was performed at 55°C for 30 s, an annealing step of nrSSU and nrLSU region was performed at 52°C for 45 s, whereas annealing step of the *tub2* gene was performed at 52°C for 30 s; an elongation step were performed at 72°C for 1 min; lastly to amplify the *tef1-α* gene, annealing step at 57°C for 1 min and an elongation step at 72°C for 1.30 min were was performed. Cycles were performed 35 times, with a final extension at 72°C for 10 min on a peqSTAR thermal cycler (PEQLAB Ltd., Fareham, UK). PCR products were checked on 1% agarose gel electrophoresis and measured quantity with NanoDrop OneC (Thermo Scientific, USA). Then, the PCR clean-up Gel Extraction NucleoSpin^®^ Gel and the PCR Clean-up Kit (Macherey-Nagel, Düren, Germany) were used to purify PCR products. Purified PCR products were sequenced by 1st BASE Company (Kembangan, Malaysia).

### Sequence alignment and phylogenetic analyses

The ITS, nrLSU, nrSSU, *tub2*, and *tef1-α* sequence data were assembled using the software Sequencher 5.4.6 (Nishimura, 2000). The consensus sequences were blasted in the BLAST search tool via NCBI website. Two datasets were prepared to construct the phylogenetic trees for clarifying the family *Herpotrichiellaceae* (**Table 1**) and genus *Cladophialophora* (**Table 2**). Multiple sequence alignment was performed by MUSCLE using MEGA 6 (Edgar, 2004) and adjusted manually in BioEdit v.7.2.5 (Hall, 2004). Phylogenetic analysis of the family *Herpotrichiellaceae* (analysis I) was carried out on the basis of only the ITS, nrLSU, and SSU sequences because the amount of available sequence data in the *tub2* and *tef1-α* genes is practically limited. To construct a phylogenetic tree of *Cladophialophora* (analysis II), five gene datasets (ITS, nrLSU, nrSSU, *tub2*, and *tef1-α*) were used. Maximum likelihood (ML) analysis was generated with 25 categories and 1,000 bootstrap (BS) replications under the GTRCAT model using the RAxML-HPC2 on XSEDE (v.8.2.12) in the CIPRES web portal (Felsenstein, 1985; Stamatakis, 2006; Miller et al., 2009). Bayesian inference (BI) analysis was performed using MrBayes v.3.2.6 (Ronquist and Huelsenbeck, 2003). Bayesian posterior probability (PP) was determined by Markov chain Monte Carlo (MCMC) sampling. Six simultaneous Markov chains were run for 5 million generations for analysis I and 2 million generations for analysis II with random initial trees, wherein every 100th generations were sampled. The first 20% of generated trees representing the burn-in phase of the analysis were discarded, whereas the remaining trees were used for calculating PP in the majority-rule consensus tree. Branches with BS support and PP values of more than or equal to 75% and 0.95, respectively, were deemed to have been substantially supported. The tree topologies were visualized in FigTree v1.4.0 (Rambaut, 2019).

**Table 1:** GenBank accession numbers of herpotrichiellaceous fungi used in the molecular phylogenetic analysis.

Species	Strains	GenBank Accession No.			References
		ITS	nrLSU	nrSSU	
*Aculeata aquatica*	MFLUCC 11-0529^T^	MG922571	MG922575	MG922579	Dong et al. (2018)
*Ac. ramosa*	CGMCC 3.16372^T^	KP174844	KP174923	KP174883	Sun et al. (2020)
*Ac. saxincola*	CGMCC 3.17315^T^	KP174843	KP174924	KP174880	Sun et al. (2020)
*Arthrocladium caudatum*	CBS 457.67^T^	LT558701	KT337443	LT558701	Nascimento et al. (2016); Réblová et al. (2016)
*Ar. fulminans*	CBS 136243^T^	KT337439	KT337444	−	Nascimento et al., 2016
*Ar. tropicale*	CBS 134926^T^	KX822543	KX822350	KX822310	Vasse et al. (2017)
*Atrokylindriopsis setulosa*	HMAS245592^T^	KP337330	KP337329	−	Ma et al. (2015)
*Bradymyces alpinus*	CCFEE 5493^T^	HG793052	GU250396	GU250354	Hubka et al. (2014)
*Br. graniticola*	CBS 140773^T^	LT558704	LT558704	LT558704	Réblová et al. (2016)
*Br. oncorhynchi*	CCF 4369^T^	HG426062	HG426063	HG426064	Hubka et al. (2014)
*Brycekendrickomyces acaciae*	CBS 124104^T^	FJ839606	FJ839641	−	Crous et al. (2009)
*Capronia acutiseta*	CBS 618.96^T^	AF050241	KF155191	AJ232942	Untereiner and Naveau (1999); Vicente et al. (2014)
*Ca. camelliae-yunnanensis*	CGMCC 3.19061^T^	MH807377	MH807378	MH807379	Phookamsak et al. (2019)
*Ca. capucina*	BBB561^T^	MH809170	MH791322	−	Sánchez et al. (2019)
*Ca. coronata*	CBS 617.96^T^	JF747040	AF050242	JN856009	Untereiner and Naveau (1999); de Hoog et al. (2011)
*Ca. dactylotricha*	CBS 604.96^T^	AF050243	KX712343	AJ232943	Untereiner and Naveau (1999); Teixeira et al. (2017)
*Ca. fungicola*	CBS 614.96^T^	KY484990	FJ358224	FJ358292	Gueidan et al. (2008)
*Ca. leucadendri*	CBS 122672^T^	EU552108	MH874754	−	Marincowitz et al. (2008); Vu et al. (2019)
*Ca. mansonii*	CBS 101.67^T^	AF050247	AY004338	X79318	Untereiner and Naveau (1999); Lumbsch et al. (2005)
*Ca. munkii*	CBS 615.96^T^	MH862601	−	−	Vu et al. (2019)
*Ca. nigerrima*	CBS 513.69	MH859363	AY605075	AY541478	Lumbsch et al. (2004); Lumbsch et al. (2005); Vu et al. (2019)
*Ca. parasitica*	CBS 123.88	AF050252	FJ358225	JN941212	Untereiner and Naveau (1999); Gueidan et al. (2008)
*Ca. pilosella*	AFTOL-ID 657	DQ826737	DQ823099	DQ823106	James et al. (2006)
*Ca. rubiginosa*	BBB 536^T^	MH809171	MH791323	−	Sánchez et al. (2019)
*Cladophialophora abundans*	CBS 126736^T^	KC776592	KC812100	−	Feng et al. (2014)
*Cl. aquatica*	MFLUCC 21-0108^T^	MT864355	MT860433	MT860446	Boonmee et al. (2021)
*Cl. arxii*	CBS 306.94^T^	EU103986	KX822320	AJ232948	Haase et al. (1999); Badali et al. (2009); Vasse et al. (2017)
*Cl. australiensis*	CBS 112793^T^	EU137331	EU035402	KX822275	Crous et al. (2007); Feng et al. (2014); Vasse et al. (2017)
*Cl. bantiana*	CBS 173.52^T^	EU103989	KF155189	AY554284	Badali et al. (2009); Vicente et al. (2014)
*Cl. boppii*	CBS 126.86^T^	MH861932	FJ358233	FJ358301	Gueidan et al. (2008); Vu et al. (2019)
*Cl. bromeliacearum*	URM 8085^T^	MW794272	MW794274	−	Nascimento et al. (2021)
*Cl. cabanerensis*	CBS 146718^T^	MN310213	MN308512	−	Crous et al. (2020a)
*Cl. carrionii*	CBS 160.54^T^	EU137266	FJ358234	FJ358302	de Hoog et al. (2007); Gueidan et al. (2008)
*Cl. chaetospira*	CBS 491.70^T^	EU035405	EU035405	KX822276	Crous et al. (2007); Vasse et al. (2017)
*Cl. devriesii*	CBS 147.84^T^	EU103985	KC809989	AJ232947	Haase et al. (1999); Badali et al. (2009); Feng et al. (2014)
*Cl. emmonsii*	CBS 979.96^T^	EU103996	−	−	Badali et al. (2009)
*Cl. eucalypti*	CBS 145551^T^	MK876380	MK876419	−	Crous et al. (2019a)
*Cl. exuberans*	CMRP1227^T^	KY680429	KY570931	−	Nascimento et al. (2017)
*Cl. floridana*	NRRL 66282^T^	AB986343	AB986343	−	Obase et al. (2016)
*Cl. immunda*	CBS 834.96^T^	MH862619	KC809990	KF155194	Feng et al. (2014); Vu et al. (2019)
*Cl. inabaensis*	EUCL1^T^	LC128795	−	−	Usui et al. (2016)
*Cl. lanosa*	KNU 16032^T^	LC387460	LC387461	−	Das et al. (2019)
*Cl. matsushimae*	MFC-1P384^T^	FN549916	FN400758	−	Koukol (2010)
*Cl. minourae*	CBS 556.83^T^	AY251087	FJ358235	FJ358303	Braun et al. (2003); Gueidan et al. (2008)
*Cl. multiseptata*	CBS 136675^T^	HG003668	HG003671	−	Crous et al. (2013)
*Cl. mycetomatis*	CBS 122637^T^	FJ385276	KX822321	KX822278	Badali et al. (2008); Vasse et al. (2017)
*Cl. nyingchiensis*	CGMCC 3.17330^T^	MG012699	MG197824	MG012728	Sun et al. (2020)
*Cl. parmeliae*	CBS 129337	JQ342180	JQ342182	−	Diederich et al. (2013)
*Cl. potulentorum*	CBS 115144^T^	DQ008141	−	−	Badali et al. (2009)
*Cl. proteae*	CBS 111667^T^	EU035411	EU035411	KJ636039	Crous et al. (2007); Gueidan et al. (2014)
*Cl. psammophila*	CBS 110553^T^	AY857517	KX712346	−	Prenafeta-Boldú et al. (2006); Teixeira et al. (2017)
*Cl. pseudocarrionii*	CBS 138591^T^	KU705827	KU705844	−	Madrid et al. (2016)
*Cl. pucciniophila*	KACC 43957^T^	JF263533	JF263534	−	Park and Shin (2011)
*Cl. recurvata*	CBS 143843^T^	LT985878	LT985879	−	Rodríguez-Andrade et al. (2019)
***Cl. rupestricola***	**SDBR-CMU446^T^**	**OP903465**	**OP903502**	**OR141860**	**This study**
***Cl. rupestricola***	**SDBR-CMU447**	**OP903466**	**OP903503**	**OR141861**	**This study**
***Cl. rupestricola***	**SDBR-CMU448**	**OP903467**	**OP903504**	**OR141862**	**This study**
*Cl. samoensis*	CBS 259.83^T^	MH861581	KC809992	KX822281	Feng et al. (2014); Vasse et al. (2017); Vu et al. (2019)
*Cl. saturnica*	CBS 118724^T^	EU103984	−	−	Badali et al. (2009)
***Cl. sribuabanensis***	**SDBR-CMU476^T^**	**OQ991178**	**OQ979608**	**OR141868**	**This study**
***Cl. sribuabanensis***	**SDBR-CMU477**	**OQ991179**	**OQ979609**	**OR141869**	**This study**
*Cl.subtilis*	CBS 122642^T^	FJ385273	KX822322	KX822283	Badali et al. (2008); Vasse et al. (2017)
*Cl. tengchongensis*	CGMCC3.15201^T^	MG012702	MG197827	MG012731	Sun et al. (2020)
***Cl. thailandensis***	**SDBR-CMU449**	**OP903468**	**OP903505**	**OR141863**	**This study**
***Cl. thailandensis***	**SDBR-CMU450**	**OP903469**	**OP903506**	**OR141864**	**This study**
***Cl. thailandensis***	**SDBR-CMU451^T^**	**OP903470**	**OP903507**	**OR141865**	**This study**
***Cl. thailandensis***	**SDBR-CMU452**	**OP903471**	**OP903508**	**OR141866**	**This study**
***Cl. thailandensis***	**SDBR-CMU453**	**OP903472**	**OP903509**	**OR141867**	**This study**
*Cl. tortuosa*	ATCC TSD-9^T^	AB986424	AB986424	−	Obase et al. (2016)
*Cl. tumbae*	JCM 28749^T^	LC192107	LC192072	−	Kiyuna et al. (2018)
*Cl. tumulicola*	JCM 28766^T^	LC192098	LC192063	−	Kiyuna et al. (2018)
*Cl. yegresii*	CBS 114405^T^	EU137322	KC809994	KX822284	de Hoog et al. (2007); Feng et al. (2014); Vasse et al. (2017)
*Epibryon interlamellare*	CBS 126286	MH863958	MH875417	−	Vu et al. (2019)
*Ep. turfosorum*	CBS 126587	MH864165	MH875627	−	Vu et al. (2019)
*Exophiala abietophila*	CBS 145038^T^	MK442581	MK442523	−	Crous et al. (2019b)
*Ex. alcalophila*	CBS 520.82^T^	MH861524	AF361051	JN856010	de Hoog et al. (2011); Vu et al. (2019)
*Ex. angulospora*	CBS 482.92^T^	MH862370	KF155190	JN856011	de Hoog et al. (2011); Vicente et al. (2014); Vu et al. (2019)
*Ex. bergeri*	CBS 353.52^T^	MH857080	FJ358240	FJ358308	Gueidan et al. (2008); Vu et al. (2019)
*Ex. bonariae*	CBS 139957^T^	JX681046	KR781083	−	Isola et al. (2016)
*Ex. brunnea*	CBS 587.66^T^	MH858890	KX712342	JN856013	de Hoog et al. (2011); Teixeira et al. (2017); Vu et al. (2019)
*Ex. castellanii*	CBS 158.58^T^	MH857734	KF928522	JN856014	de Hoog et al. (2011); Attili-Angelis et al. (2014); Vu et al. (2019)
*Ex. crusticola*	CBS 119970^T^	MH863070	KF155180	KF155199	Vicente et al. (2014); Vu et al. (2019)
*Ex. dermatitidis*	CBS 207.35^T^	MH855649	KF928508	−	Attili-Angelis et al. (2014); Vu et al. (2019)
*Ex. encephalarti*	CBS 128210^T^	HQ599588	HQ599589	−	de Hoog (2010)
*Ex. eucalypti*	CBS 142069	KY173411	KY173502	−	Crous et al. (2016)
*Ex. eucalypticola*	CBS 143412^T^	MH107891	MH107938	−	Crous et al. (2018a)
*Ex. jeanselmei*	CBS 507.90^T^	AY156963	KJ930161	FJ358310	Vitale and de Hoog (2002); Gueidan et al. (2008)
*Ex. lecanii-corni*	CBS 123.33^T^	MH855383	FJ358243	FJ358311	Gueidan et al. (2008); Vu et al. (2019)
*Ex. mesophila*	CBS 402.95^T^	MH862536	KX712349	JN856016	de Hoog et al. (2011); Teixeira et al. (2017); Vu et al. (2019)
*Ex. nidicola*	CBS 138589^T^	MG701055	MG701056	−	Crous et al. (2018b)
*Ex. nishimurae*	CBS 101538^T^	JX473274	KX712351	KX822288	Woo et al. (2013); Teixeira et al. (2017); Vasse et al. (2017)
*Ex. oligosperma*	CBS 725.88^T^	AY163551	FJ358245	AY554287	Haase et al. (1999); de Hoog et al. (2003); Gueidan et al. (2008)
*Ex. opportunisticica*	CBS 109811^T^	KF928437	KF928501	−	Attili-Angelis et al. (2014)
*Ex. pisciphila*	CBS 537.73^T^	DQ826739	AF050272	DQ823108	Untereiner and Naveau (1999); James et al. (2006)
*Ex. placitae*	CBS 121716^T^	MH863143	MH874694	−	Vu et al. (2019)
*Ex. quercina*	CBS 146024^T^	MT223797	MT223892	−	Crous et al. (2020b)
*Ex.radicis*	P2854^T^	KT099204	KT723448	KT723453	Glynou et al. (2016); Maciá-Vicente et al. (2016)
*Ex. salmonis*	CBS 157.67^T^	JF747137	AY213702	EF413608	Rakeman et al. (2005); Geiser et al. (2006); de Hoog et al. (2011)
*Ex. siamensis*	SDBR-CMU417^T^	ON555811	−	ON555826	Thitla et al. (2022)
*Ex. siamensis*	SDBR-CMU418	ON555812	−	ON555827	Thitla et al. (2022)
*Ex. sideris*	CBS 121818^T^	HQ452311	−	HQ441174	Seyedmousavi et al. (2011)
*Ex. spinifera*	CBS 899.68^T^	MH859248	MH870977	−	Vu et al. (2019)
*Fonsecaea brasiliensis*	CBS 119710^T^	JN173784	KF155183	KF155203	Vicente et al. (2012); Vicente et al. (2014)
*F. erecta*	CBS 125763^T^	KC886414	KF155186	KF155210	Vicente et al. (2014)
*F. monophora*	CBS 102243^T^	EU938579	FJ358247	FJ358315	Gueidan et al. (2008); Najafzadeh et al. (2009)
*F. multimorphosa*	CBS 980.96^T^	JF267657	KF155188	JF433950	Najafzadeh et al. (2011); Vicente et al. (2014)
*F. pedrosoi*	CBS 271.37^T^	AB114127	KJ930166	AY554290	Haase et al. (1999); Tanabe et al. (2004); Li et al. (2017)
*Knufia cryptophialidica*	DAOM 216555^T^	JN040501	JN040500	EF137364	Scott et al. (2007); Tsuneda et al. (2011)
*K. epidermidis*	CBS 120353 ^T^	EU730589	FJ355954	FJ355953	Li et al. (2008)
*K. marmoricola*	CBS 139726 ^T^	KP791775	KR781063	−	Isola et al. (2016)
*K. mediterranea*	CBS 139721 ^T^	KP791791	KR781078	−	Isola et al. (2016)
*K. petricola*	CBS 726.95^T^	KC978746	KC978741	KC978739	Nai et al. (2013)
*Lithohypha aloicola*	CPC 35996^T^	MN562103	MN567611	−	Crous et al. (2019c)
*L. catenulata*	CGMCC 3.14885^T^	JN650519	KP174917	KP174911	Sun et al. (2020)
*L. guttulata*	CBS 139723^T^	KP791773	KR781061	−	Isola et al. (2016)
*Marinophialophora garethjonesii*	KUMCC 16-0066^T^	KY305175	KY305177	KY305179	Li et al. (2018)
*Melanoctona tectonae*	MFLUCC 12-0389^T^	KX258778	KX258779	KX258780	Tian et al. (2016)
*Metulocladosporiella musae*	CBS 161.74^T^	DQ008137	DQ008161	−	Ávila et al. (2005)
*Met. musicola*	CBS 110960^T^	DQ008127	DQ008153	−	Ávila et al. (2005)
*Minimelanolocus clavatus*	DLU 3022^T^	MT271774	MT271772	MT271777	Wan et al. (2021)
*Mi. yunnanensis*	MFLUCC 16-0764^T^	KX258781	KX258782	KX258783	Tian et al. (2016)
*Neosorocybe pini*	CBS 146085^T^	MT223824	MT223916	−	Crous et al. (2020b)
*Neostrelitziana acaciigena*	CBS 139903^T^	KR476730	KR476765	−	Crous et al. (2015)
***Petriomyces obovoidisporus***	**SDBR-CMU478^T^**	**OQ991180**	**OQ979610**	**OR141870**	**This study**
***Pe. obovoidisporus***	**SDBR-CMU479**	**OQ991181**	**OQ979611**	**OR141871**	**This study**
*Phialophora chinensis*	CBS 140326^T^	KF881964	KJ930093	KM658060	Li et al. (2017)
*Phia. ellipsoidea*	CBS 286.47^T^	AF050282	AF050282	−	Untereiner and Naveau (1999)
*Phia. expanda*	BMU 02323^T^	KF881937	MH878677	−	Li et al. (2017); Vu et al. (2019)
*Phia. macrospora*	CBS 273.37^T^	AF050281	AF050281	−	Untereiner and Naveau (1999)
*Phia. verrucosa*	CBS 140325^T^	KF881960	KJ930073	KM658059	Li et al. (2017)
*Rhinocladiella amoena*	CBS 138590^T^	KU705840	KU705857	−	Madrid et al. (2016)
*Rhin. anceps*	CBS 181.65^T^	EU041805	EU041862	AY554292	Haase et al. (1999); Arzanlou et al. (2007)
*Rhin. atrovirens*	CBS 317.33^T^	AB091215	MH866906	−	Abliz et al. (2003); Vu et al. (2019)
*Rhin. basitona*	CBS 101460^T^	EU041806	EU041863	−	Arzanlou et al. (2007)
*Rhin. coryli*	CBS 141407^T^	KX306768	KX306793	−	Hernández-Restrepo et al. (2016)
*Rhin. mackenziei*	CBS 650.93^T^	AY857540	AF050288	−	Untereiner and Naveau (1999); Prenafeta-Boldú et al. (2006)
*Rhin. phaeophora*	CBS 496.78^T^	EU041811	EU041868	EF137366	Arzanlou et al. (2007); Scott et al. (2007)
*Rhin. pyriformis*	CBS 469.94^T^	MH862476	−	−	Vu et al. (2019)
*Rhin. quercus*	CBS 141448^T^	KX306769	KX306794	−	Hernández-Restrepo et al. (2016)
*Rhin. similis*	CBS 111763^T^	EF551461	−	−	Zeng and de Hoog (2008)
*Sorocybe oblongispora*	DAOMC 251618^T^	MN114116	MN114118	−	Crous et al. (2019c)
*So. resinae*	DAOM 239134	EU030275	EU030277	−	Seifert et al. (2007)
*Strelitziana africana*	CBS 120037^T^	DQ885895	DQ885895	−	Arzanlou and Crous (2006)
*St. albiziae*	CBS 126497^T^	HQ599584	HQ599585	−	de Hoog (2010).
*St. australiensis*	CBS 124778^T^	GQ303295	GQ303326	−	Cheewangkoon et al. (2009)
*Thysanorea cantrelliae*	CBS 145909^T^	MN794376	MN794353	MN794382	Hernández-Restrepo et al. (2010)
*Th. papuana*	CBS 212.96^T^	MH862572	MH874198	−	Vu et al. (2019)
*Th. seifertii*	CBS 145910^T^	MN794377	MN794354	MN794383	Hernández-Restrepo et al. (2010)
*Th. thailandensis*	MFLUCC 15-0971^T^	MG922573	MG922577	MG922581	Dong et al. (2018)
*Th. yunnanensis*	MFLUCC 15-0414^T^	KR215607	KR215612	KR215617	Liu et al. (2015)
*Trichomerium dioscoreae*	CBS 138870^T^	KP004468	KP004496	−	Crous et al. (2014)
*Tr. foliicola*	MFLUCC 10-0078^T^	JX313655	JX313661	−	Chomnunti et al. (2011)
*Tr. gloeosporum*	MFLUCC 10-0087^T^	JX313656	JX313662	−	Chomnunti et al. (2011)
*Uncispora sinensis*	YMF 1.03683^T^	KU173860	KU558914	KU558913	Yang et al. (2011)
*U. wuzhishanensis*	YMF 1.04080^T^	KU173859	KU558912	KU558911	Liu et al. (2018)
*Valentiella maceioensis*	BSS 376	MZ042488	MZ042486	−	Bezerra et al. (2022)
*Va. maceioensis*	CBS 141892	KY305141	KX348014	−	Bezerra et al. (2022)
*Veronaea botryosa*	CBS 254.57^T^	MH857711	MH869255	JN856021	de Hoog et al. (2011); Vu et al. (2019)
*Ve. japonica*	CBS 776.83^T^	EU041818	EU041875	−	Arzanlou et al. (2007)

Species obtained in this study are in bold. Superscript “T” represents ex-type species. “−” represents the absence of sequence data in GenBank database.

**Table 2:** GenBank accession numbers of *Cladophialophora* in the family *Herpotrichiellaceae* used in the molecular phylogenetic analysis.

Species	Strains	GenBank Accession No.					References
		ITS	nrLSU	nrSSU	*tub2*	*tef1-α*	
*Cladophialophora abundans*	CBS 126736^T^	KC776592	KC812100	−	−	−	Feng et al. (2014)
*Cl. abundans*	MFLUCC 21-0105	MT864354	MT860432	−	−	−	Boonmee et al. (2021)
*Cl. aquatica*	MFLUCC 21-0108^T^	MT864355	MT860433	−	−	−	Boonmee et al. (2021)
*Cl. arxii*	CBS 306.94^T^	EU103986	KX822320	AJ232948	−	EU140593	Haase et al. (1999); Badali et al. (2009); Vasse et al. (2017)
*Cl. arxii*	IFM 52022	AB109181	LT883516	−	−	−	Abliz et al. (2004)
*Cl. australiensis*	CBS 112793^T^	EU035402	EU035402	KX822275	–	−	Crous et al. (2007); Vasse et al. (2017)
*Cl. bantiana*	CBS 173.52^T^	EU103989	KF155189	AY554284	−	EU140585	Haase et al. (1999); Badali et al. (2009); Vicente et al. (2014)
*Cl. bantiana*	CBS 119719	EU103991	−	−	−	EU140589	Badali et al. (2009)
*Cl. boppii*	CBS 110029	EU103998	−	−	−	EU140597	Badali et al. (2009)
*Cl. boppii*	CBS 126.86^T^	EU103997	FJ358233	FJ358301	−	EU140596	Gueidan et al. (2008); Badali et al. (2009)
*Cl. bromeliacearum*	URM 8085^T^	MW794272	MW794274	−	MW810487	−	Nascimento et al. (2021)
*Cl. bromeliacearum*	FCCUFG 04	MW794273	MW794275	−	MW810488	−	Nascimento et al. (2021)
*Cl. cabanerensis*	CBS 146718^T^	MN310213	MN308512	−	−	−	Crous et al. (2020a)
*Cl. cabanerensis*	P6479	–	MN308510	−	−	−	Crous et al. (2020a)
*Cl. carrionii*	CBS 114393	EU137268	KF928516	−	EU137151	EU137212	de Hoog et al. (2007); Attili-Angelis et al. (2014)
*Cl. carrionii*	CBS 160.54^T^	EU137266	KF928517	FJ358302	EU137201	EU137210	de Hoog et al. (2007); Gueidan et al. (2008); Attili-Angelis et al. (2014)
*Cl. chaetospira*	CBS 491.70^T^	EU035405	EU035405	KX822276	−	−	Crous et al. (2007); Vasse et al. (2017)
*Cl. chaetospira*	CBS 114747	EU035403	EU035403	−	KF928578	−	Crous et al. (2007); Attili-Angelis et al. (2014)
*Cl. devriesii*	CBS 147.84^T^	EU103985	KC809989	AJ232947	−	EU140595	Badali et al. (2009); Vicente et al. (2014)
*Cl. devriesii*	CBS 127019	MH864392	MH875832	−	−	−	Vu et al. (2019)
*Cl. emmonsii*	CBS 640.96	EU103995	KC809995	−	−	EU140584	Feng et al. (2014); Vicente et al. (2014)
*Cl. emmonsii*	CBS 979.96^T^	EU103996	−	−	−	EU140583	Vicente et al. (2014)
*Cl. exuberans*	CMRP1219	KY680430	KY570930	−	KY689827	−	Nascimento et al. (2017)
*Cl. exuberans*	CMRP1227^T^	KY680429	KY570931	−	KY689826	−	Nascimento et al. (2017)
*Cl. floridana*	NRRL 66282^T^	AB986343	AB986343	−	−	−	Obase et al. (2016)
*Cl. floridana*	NRRL 66283	AB986344	AB986344	−	−	−	Obase et al. (2016)
*Cl. immunda*	CBS 110551	FJ385274	−	−	EU137207	EU137261	Badali et al. (2008)
*Cl. immunda*	CBS 834.96^T^	EU137318	KC809990	KF155194	EU137203,	EU137257	Badali et al. (2008); Feng et al. (2014); Vicente et al. (2014)
*Cl. inabaensis*	EUCL1^T^	LC128795	LC128795	−	−	−	Usui et al. (2016)
*Cl. lanosa*	KNU16-032^T^	LC387460	LC387461	−	−	−	Das et al. (2019)
*Cl. matsushimae*	MFC-1P384^T^	FN549916	FN400758	−	−	−	Koukol (2010)
*Cl. matsushimae*	CMRP1198	KY680416	−	−	−	−	Nascimento et al. (2017)
*Cl. minourae*	CBS 556.83^T^	AY251087	FJ358235	FJ358303	−	EU140598	Braun et al. (2003); Badali et al. (2008); Gueidan et al. (2008)
*Cl. multiseptata*	CBS 136675^T^	HG003668	HG003671	−	−	−	Crous et al. (2013)
*Cl. multiseptata*	dH21520	−	KX712345	−	−	−	Teixeira et al. (2017)
*Cl. mycetomatis*	CBS 122637^T^	FJ385276	KX822321	KX822278	−	−	Badali et al. (2008); Vasse et al. (2017)
*Cl. mycetomatis*	CBS 454.82	EU137293	KC809991	KX822279	EU137176	EU137235	Badali et al. (2008); Feng et al. (2014); Vasse et al. (2017)
*Cl. nyingchiensis*	CGMCC 3.17329	MG012700	MG197825	MG012729	MG012748	MG012707	Sun et al. (2020)
*Cl. nyingchiensis*	CGMCC 3.17330^T^	MG012699	MG197824	MG012728	MG012747	MG012706	Sun et al. (2020)
*Cl. parmeliae*	CBS 129337	JQ342180	JQ342182	−	−	−	Diederich et al. (2013)
*Cl. parmeliae*	CBS 132232	−	JX081671	−	−	−	Diederich et al. (2013)
*Cl. potulentorum*	CBS 115144^T^	DQ008141	−	−	−	−	Crous et al. (2007)
*Cl. potulentorum*	CBS 112222	EU035409	−	−	−	−	Badali et al. (2008)
*Cl. psammophila*	CBS 110553^T^	AY857517	KX712346	−	−	−	Prenafeta-Boldú et al. (2006); Teixeira et al. (2017)
*Cl. pseudocarrionii*	CBS 138591^T^	KU705827	KU705844	−	−	−	Madrid et al. (2016)
*Cl. recurvata*	CBS 143843^T^	LT985878	LT985879	−	−	−	Rodríguez-Andrade et al. (2019)
***Cl. rupestricola***	**SDBR-CMU446^T^**	**OP903465**	**OP903502**	**OR141860**	**OR139230**	**OP923695**	**This study**
***Cl. rupestricola***	**SDBR-CMU447**	**OP903466**	**OP903503**	**OR141861**	**OR139231**	**OP923696**	**This study**
***Cl. rupestricola***	**SDBR-CMU448**	**OP903467**	**OP903504**	**OR141862**	**OR139232**	**OP923697**	**This study**
*Cl. samoensis*	CBS 259.83^T^	EU137291	KC809992	KX822281	EU137174	EU137233	Badali et al. (2008); Feng et al. (2014); Vasse et al. (2017)
*Cl. saturnica*	CBS 102230	AY857508	KC809993	KX822282	−	EU140600	Prenafeta-Boldu et al. (2006); Badali et al. (2008); Feng et al. (2014); Vasse et al. (2017)
*Cl. saturnica*	CBS 118724^T^	EU103984	−	−	−	EU140602	Badali et al. (2009)
***Cl. sribuabanensis***	**SDBR-CMU476^T^**	**OQ991178**	OQ979608	**OR141868**	**OR139238**	**OR139226**	**This study**
***Cl. sribuabanensis***	**SDBR-CMU477**	**OQ991179**	**OQ979609**	**OR141869**	**OR139239**	**OR139227**	**This study**
*Cl. subtilis*	CBS 122642^T^	FJ385273	KX822322	KX822283	−	−	Badali et al. (2008); Vasse et al. (2017)
*Cl. tengchongensis*	CGMCC3.15201^T^	MG012702	MG197827	MG012731	MG012750	MG012709	Sun et al. (2020)
*Cl. tengchongensis*	CGMCC3.15204	MG012703	MG197828	MG012732	MG012751	MG012710	Sun et al. (2020)
***Cl. thailandensis***	**SDBR-CMU449**	**OP903468**	**OP903505**	**OR141863**	**OR139233**	**OP923698**	**This study**
***Cl. thailandensis***	**SDBR-CMU450**	**OP903469**	**OP903506**	**OR141864**	**OR139234**	**OP923699**	**This study**
***Cl. thailandensis***	**SDBR-CMU451^T^**	**OP903470**	**OP903507**	**OR141865**	**OR139235**	**OP923700**	**This study**
***Cl. thailandensis***	**SDBR-CMU452**	**OP903471**	**OP903508**	**OR141866**	**OR139236**	**OP923701**	**This study**
***Cl. thailandensis***	**SDBR-CMU453**	**OP903472**	**OP903509**	**OR141867**	**OR139237**	**OP923702**	**This study**
*Cl. tortuosa*	ATCC TSD-9^T^	AB986424	AB986424	−	−	−	Obase et al. (2016)
*Cl. tumbae*	JCM 28749^T^	LC192107	LC192072	−	−	−	Kiyuna et al. (2018)
*Cl. tumbae*	JCM 28753	LC192108	LC192073	−	−	−	Kiyuna et al. (2018)
*Cl. tumulicola*	JCM 28766^T^	LC192098	LC192063	−	−	−	Kiyuna et al. (2018)
*Cl. tumulicola*	JCM 28758	LC192094	LC192059	−	−	−	Kiyuna et al. (2018)
*Cl. yegresii*	CBS 114405^T^	EU137322	KX822323	KX822284	EU137209	EU137262	de Hoog et al. (2007); Vasse et al. (2017)
*Cl. yegresii*	CBS 114406	EU137323	−	−	EU137208	EU137263	de Hoog et al. (2007)
*Bradymyces alpinus*	CCFEE 5493^T^	HG793052	GU250396	GU250354	LN589970	−	Hubka et al. (2014)
*Br. oncorhynchi*	CCF 4369^T^	HG426062	HG426063	HG426064	HG426060	−	Hubka et al. (2014)

Species obtained in this study are in bold. Superscript “T” represents ex-type species. “−” represents the absence of sequence data in GenBank database.

## Results

### Fungal isolation, morphological study, and growth temperature

A total of 12 fungal strains were obtained from different rock samples. Ten fungal strains (SDBR-CMU446, SDBR-CMU447, SDBR-CMU448, SDBR-CMU449, SDBR-CMU450, SDBR-CMU451, SDBR-CMU452, SDBR-CMU453, SDBR-CMU476, and SDBR-CMU477) exhibited similar characteristics by appearing one-celled and hyaline and by forming conidial chains. Initially, these 10 fungal strains were identified as *Cladophialophora* species according to their micromorphological features. However, they were divided into three different groups on the basis of their colony characteristics on culture media, micromorphological, and growth temperature profiles (**Table 3**). In addition, two fungal strains (SDBR-CMU478 and SDBR-CMU479) appeared similar in their characteristics with produced sympodial conidial formation on conidiogenous loci and by appearing one-celled, hyaline to subhyaline, and obovoidal conidia. On the basis of morphological characteristics, these two fungal strains could not be assigned to any genera. Therefore, multi-gene phylogenetic analyses were used to identify their species-level and phylogenetic placement.

**Table 3:** Colony diameter of obtained fungi cultured on MEA at various temperatures for 28 days in the dark.

Fungal taxa	Colony diameter (mm)*						
	10°C	15°C	20°C	25°C	28°C	30°C	35°C
*Cladophialophora rupestricola*							
SDBR-CMU446	−	7.58 ± 0.67	7.83 ± 0.72	10.56 ± 1.85	10.67 ± 0.98	10.75 ± 0.62	8.25 ± 0.62
SDBR-CMU447	−	6.92 ± 0.29	7.58 ± 0.67	10.17 ± 1.62	10.25 ± 0.87	10.50 ± 0.67	7.17 ± 0.58
SDBR-CMU448	−	7.25 ± 0.87	8.08 ± 1.08	9.50 ± 0.92	9.75 ± 0.97	9.83 ± 0.58	7.33 ± 0.78
*Cl. thailandensis*							
SDBR-CMU449	6.67 ± 0.52	13.67 ± 0.52	18.50 ± 0.55	28.75 ± 1.60	34.33 ± 0.52	22.33 ± 0.82	8.83 ± 0.41
SDBR-CMU450	6.12 ± 0.44	13.82 ± 0.72	17.87 ± 0.94	27.92 ± 1.22	32.36 ± 0.71	21.74 ± 0.65	8.79 ± 0.63
SDBR-CMU451	6.25 ± 0.45	16.67 ± 1.15	18.58 ± 0.67	26.22 ± 1.35	31.00 ± 0.82	23.00 ± 0.60	8.92 ± 0.29
SDBR-CMU452	6.45 ± 0.61	15.78 ± 1.12	18.37 ± 0.95	28.52 ± 1.34	33.74 ± 0.64	22.26 ± 0.67	8.11 ± 0.74
SDBR-CMU453	6.42 ± 0.51	14.33 ± 1.56	18.75 ± 0.87	28.06 ± 1.06	33.17 ± 0.94	25.50 ± 1.68	8.50 ± 0.80
*Cl. sribuabanensis*							
SDBR-CMU476	−	5.33 ± 0.49	8.50 ± 0.71	12.89 ± 0.76	17.17 ± 1.04	14.06 ± 0.94	−
SDBR-CMU477	−	5.44 ± 0.51	8.61 ± 0.61	13.06 ± 0.94	17.28 ± 1.02	13.94 ± 0.94	−
*Petriomyces obovoidisporus*							
SDBR-CMU478	7.00 ± 0.43	10.17 ± 0.39	12.92 ± 0.90	17.50 ± 0.86	17.33 ± 0.65	7.58 ± 0.90	−
SDBR-CMU479	7.08 ± 0.51	9.67 ± 0.49	12.25 ± 0.97	17.28 ± 0.83	17.33 ± 0.98	7.42 ± 0.79	−

*The results are average colony diameter ± standard deviation, and “−“ represents no growth.

The observation of fungal growth at various temperatures (4°C−40°C) revealed that temperature had a strong influence on the fungal growth of the obtained fungi. The average colony diameter of each fungal strain is presented in **Table 3**. The results indicate that all 12 fungal strains could not grow at 4°C, 37°C, and 40°C. Three strains (SDBR-CMU446, SDBR-CMU447, and SDBR-CMU448) could not grow at 10°C but grew at temperatures ranging from 25°C to 30°C. Five strains (SDBR-CMU449, SDBR-CMU450, SDBR-CMU451, SDBR-CMU452, and SDBR-CMU453) exhibited the highest average colony diameter at 28°C. Two strains (SDBR-CMU476 and SDBR-CMU477) could not grow at 10°C and 35°C, yet they showed the greatest average colony diameter at 28°C. Two fungal strains (SDBR-CMU478 and SDBR-CMU479) grew at temperatures ranging from 10°C to 30°C and grew well at 28°C.

### Phylogenetic study

**Figure 1:**
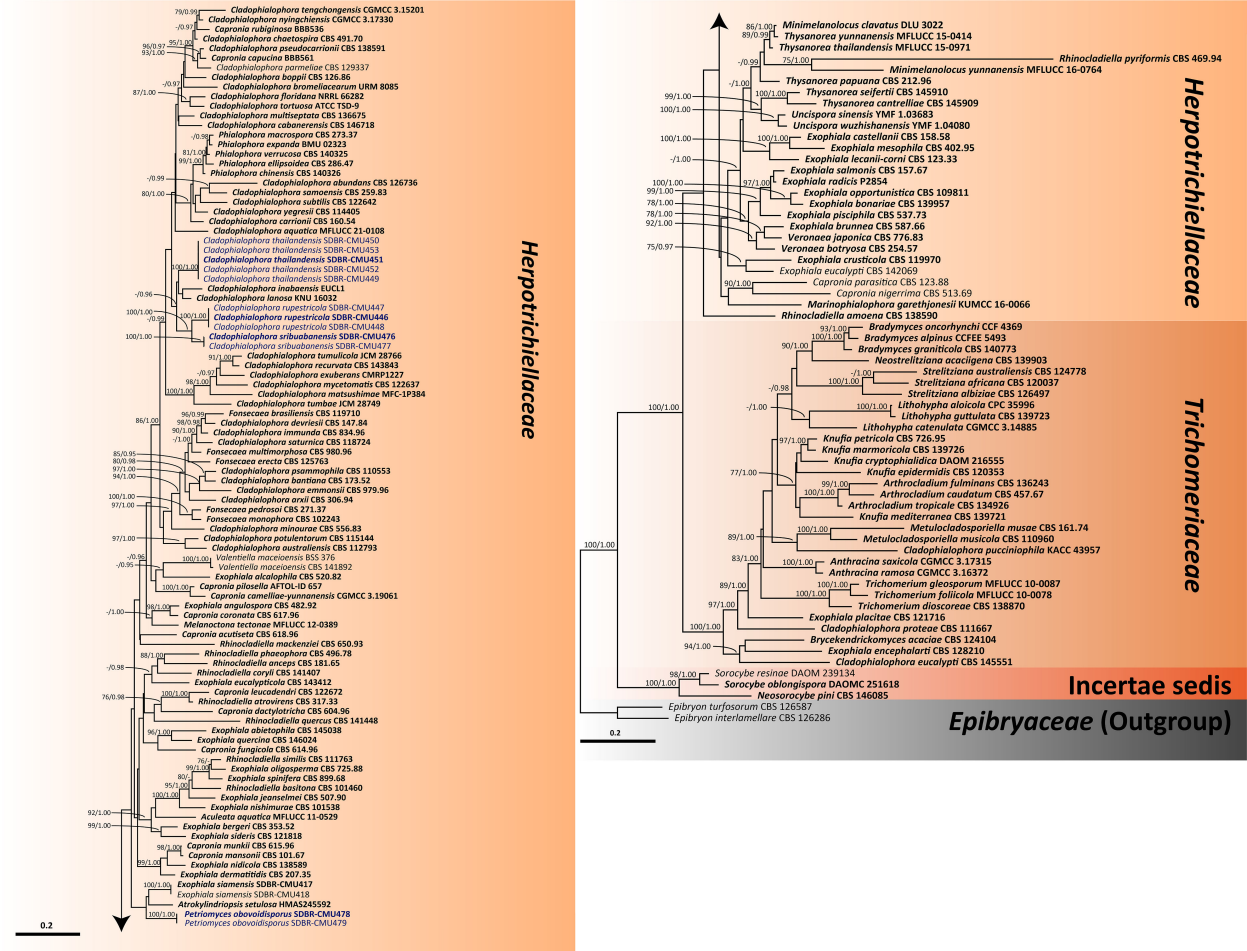
Phylogenetic tree generated from maximum likelihood analysis of 159 fungal strains based on a combined ITS, nrLSU, and nrSSU sequence dataset. *Epibryon interlamellare* CBS 126286 and *Ep. turfosorum* CBS 126587 were used as the outgroup. The numbers above branches show bootstrap percentages (left) and Bayesian posterior probabilities (right). Bootstrap values ≥75% and Bayesian posterior probabilities ≥0.95 are shown. The scale bar reflects the estimated number of nucleotide substitutions per site. The newly generated sequences are in blue. The ex-type species are in bold.

For analysis I, a phylogenetic tree of the family *Herpotrichiellaceae* was constructed from a combined ITS, nrLSU, and nrSSU sequence dataset. The dataset comprised 159 sequence strains from representatives in the families *Herpotrichiellaceae* and *Trichomeriaceae*, including the new strains that were proposed in this study. *Epibryon interlamellare* CBS 126286 and *Ep. turfosorum* CBS 126587 (famliy *Epibryaceae*) were selected as the outgroup. The concatenated dataset comprised 3,022 positions (ITS, 1−928 base pair (bp); nrLSU, 929−1,842 bp; and nrSSU, 1,843−3,022 bp) including gaps. RAxML analysis of the integrated dataset yielded the best scoring tree with a final ML optimization likelihood value of −45,154.8753. The matrix contained 1,610 distinct alignment patterns with 38.67% undetermined characters or gaps. The estimated base frequencies were recorded as follows: A = 0.2716, C = 0.2009, G = 0.2559, and T = 0.2714; whereas the substitution rates were established as AC = 0.9883, AG = 1.7181, AT = 0.9613, CG = 0.7406, CT = 2.9999, and GT = 1.0000. The gamma distribution shape parameter alpha value was equal to 0.1267, whereas the tree length was equal to 17.4438. At the end of the total MCMC generations, the final average standard deviation of the split frequencies was calculated to be 0.009866 through BI analysis. According to the topological results, ML and BI phylogenetic analyses produced similar topologies. Therefore, only the phylogenetic tree constructed from the ML analysis is shown in **Figure 1**. The phylogenetic tree clearly separates the family *Herpotrichiellaceae* from the family *Trichomeriaceae* with strong support (100% BS and 1.00 PP). In this study, all fungal strains obtained belonged to the family *Herpotrichiellaceae* and were separated from the previously known species (**Figure 1**). *Cladophiophora rupestricola* (SDBR-CMU446, SDBR-CMU447, and SDBR-CMU448) forms a distinct lineage sister clade to *Cl. sribuabanensis* (SDBR-CMU476 and SDBR-CMU477) with 100% BS and 1.00 PP support values. Accordingly, five fungal strains (SDBR-CMU449, SDBR-CMU450, SDBR-CMU451, SDBR-CMU452, and SDBR-CMU453) of *Cl. thailandensis* form a distinct lineage closely related to *Cl. inabaensis* (EUCL1) and *Cl. lanosa* (KNU 16032). In addition, two fungal strains of *Petriomyces obovoidisporus* (SDBR-CMU478 and SDBR-CMU479) formed a well-resolved clade (100% BS and 1.00 PP; **Figure 1**) in *Herpotrichiellaceae*, with *Atrokylindriopsis setulose* (HMAS245592) and *Exophiala siamensis* (SDBR-CMU417) as the sister clade.

In our phylogenetic tree for clarifying the family *Herpotrichiellaceae* (**Figure 1**), 15 previously known herpotrichiellaceous genera (*Aculeata*, *Atrokylindriopsis*, *Capronia*, *Cladophialophora*, *Exophiala*, *Fonsecaea*, *Marinophialophora*, *Melanoctona*, *Minimelanolocus*, *Phialophora*, *Rhinocladiella*, *Thysanorea*, *Uncispora*, *Valentiella*, and *Veronaea*) and a new genus (*Petriomyces*) identified in this study were assigned to the *Herpotrichiellaceae* clade. The genera *Brycekendrickomyces* and *Metulocladosporiella* were placed in the *Trichomeriaceae* clade. The clade of genera *Neosorocybe* and *Sorocybe* was clearly separated from the clade of the families *Herpotrichiellaceae* and *Trichomeriaceae* with strong support of 100% BS and 1.00 PP (**Figure 1**).

For analysis II, the phylogenetic placement of the genus *Cladophialophora* within *Herpotrichiellaceae* was established by combining five gene sequence datasets (ITS, nrLSU, nrSSU, *tub2*, and *tef1-α*) from a total of 71 taxa. The concatenated dataset comprised 3,931 positions including gaps (ITS, 1−692 bp; nrLSU, 693−1,512 bp; nrSSU, 1,513−3,185 bp; *tub2*, 3,186−3,698; and *tef1-α*, 3,699−3,931 bp). RAxML analysis of the integrated dataset yielded the best scoring tree with a final ML optimization likelihood value of −21,115.8911. The matrix contained 1,300 distinct alignment patterns with 48.15% undetermined characters or gaps. The estimated base frequencies were recorded as follows: A = 0.2256, C = 0.2775, G = 0.2336, and T = 0.2632; whereas the substitution rates were established as AC = 1.0546, AG = 2.0969, AT = 0.9937, CG = 0.5923, CT = 3.1988, and GT = 1.0000. The gamma distribution shape parameter alpha value was equal to 0.4693, whereas the tree length was equal to 4.6134. The final average standard deviation of split frequencies at the end of total MCMC generations was calculated as 0.007796 through the BI analysis. The phylogram demonstrated that all three new species of *Cladophialophora* discovered in this study were distinctly separate from the previously known species. *Cladophialophora rupestricola* and *Cl. sribuabanensis* remained a sister group with strong support (100% BS and 1.00 PP), which was consistent with the findings of analysis I. Furthermore, *Cl. thailandensis* was a distinct lineage that is still related to *Cl. inabaensis* and *Cl. lanosa*.

### Taxonomic descriptions

#### New genus

*Petriomyces*, T. Thitla and N. Suwannarach, gen. nov.

MycoBank number: MB849334

Etymology: “*Petriomyces*” refers to the fungus that dwells in rocks.

*Mycelium*: hyaline, smooth, branched, and septate hyphae. *Conidiophores*: semi-micronematous, arising vertically from creeping hyphae at right angles, straight, branched, subhyaline to pale brown, smooth, thin-walled, and septate. *Conidiogenous cells*: integrated, intercalary or terminal, sympodial, polyblastic, subconspicuous to conspicuous conidiogenous loci, subcylindrical, subdenticulate, smooth, and thin-walled. *Conidia*: obovoid or pyriform, aseptate, hyaline to subhyaline, smooth, and hilum conspicuous.

Habitat and distribution: sandstone on natural forest; known from Sukhothai province, Thailand.

Type species: *Petriomyces obovoidisporus*, T. Thitla, J. Kumla, and N. Suwannarach

**Figure 2:**
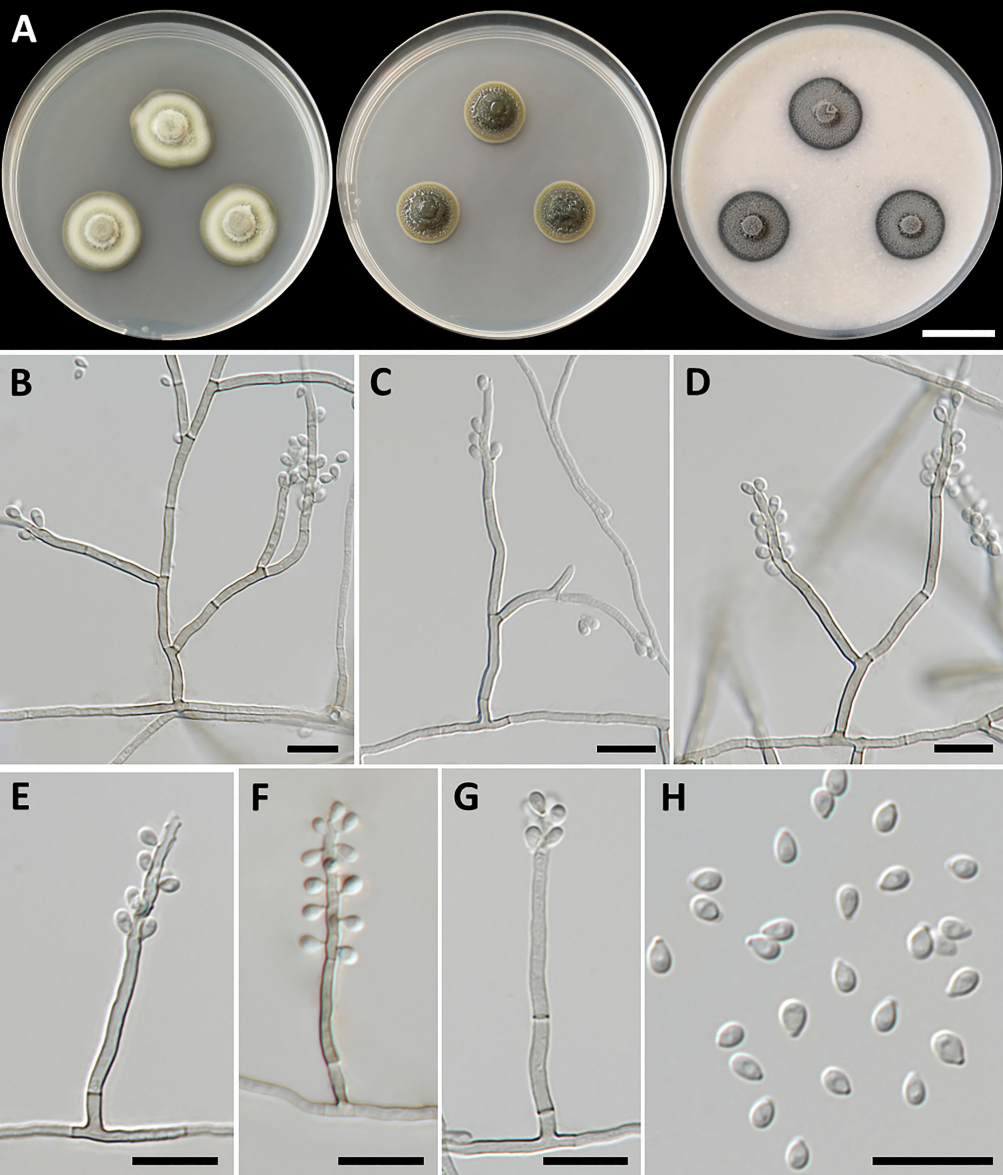
*Petriomyces obovoidisporus* (SDBR-CMU478, ex-type). **(A)** Colonies at 25°C for 28 days on PDA, MEA, and OA, respectively. **(B–G)** Conidiophore, conidiogenous cells, and conidia. **(B–H)** Conidia. Scale bars: **(A)** 2 cm and **(B–G)** 10 µm.

*Petriomyces obovoidisporus*, T. Thitla, J. Kumla, and N. Suwannarach, sp. nov. (**Figure 2**)

MycoBank number: MB849367

Etymology: “*obovoidisporus*” refers to the obovoid shape of the conidia.

Holotype: Thailand, Sukhothai province, Si Satchanalai District, 17°32′58″N 99°29′49″E, elevation at 153 m, isolated from the sandstone of natural forest, June 2021, T. Thitla; dried culture, SDBR-SKT3-6; ex-type culture, SDBR-CMU478.

GenBank number: OQ991180 (ITS), OQ979610 (nrLSU), OR141870 (nrSSU), OR139228 (*tef1-α*), and OR139240 (*tub2*)

Culture characteristics: Colonies on PDA at 25°C: attaining 20-mm to 25-mm diameter in 28 days, restricted, circular, flat, slimy, and grayish green to dull green with grayish green and entire margin; dark green in reverse; on MEA: attaining 16-mm to 20-mm diameter, restricted, circular, convex, slimy, and dark green with grayish green and entire margin; reverse grayish green to deep green; and on OA: attaining 18-mm to 22-mm diameter, restricted, circular, flat, velvety, and greenish gray with black and entire margin; reverse greenish gray. No diffusible pigment produced on any media. *Submerged hyphae* smooth, thin-walled, hyaline to light brown, 1-µm to 2-µm wide, and aerial hyphae subhyaline to pale brown with branched hyphae. *Conidiophores*: semi-micronematous, arising vertically from hyphae at right angles, straight, sometimes branched, thin-walled, subhyaline to pale brown, 1−3 septate, cylindrical, and 8−35.5 µm × 1−2 µm (mean = 18.3 µm × 1.5 µm, *n* = 30). *Conidiogenous cell*: integrated, intercalary or terminal, polyblastic, subconspicuous to conspicuous conidiogenous loci, subcylindrical, subdenticulate, smooth, thin-walled, cylindrical, slightly paler than conidiophore, and 13−65 µm × 1−2 µm (mean = 27.3 µm × 1.5 µm, *n* = 30). *Conidia*: one-celled, smooth, hyaline to subhyaline, obovoid or pyriform, 2−4 µm × 1−2 µm (mean = 2.8 µm × 1.7 µm, *n* = 50), and hilum conspicuous. *Chlamydospores* and sexual morph were not produced on media.

Growth temperature: minimum at 10°C, optimum at the range of 25°C−28°C, maximum at 30°C, and no growth at 4°C and 35°C

Additional specimens examined: Thailand, Sukhothai province, Si Satchanalai District, 17°32′58″N 99°29′49″E, elevation at 153 m, isolated from the rock of natural forest, June 2021, T. Thitla: SDBR-CMU479 [GenBank number: OQ991181 (ITS), OQ979611 (nrLSU), OR141871 (nrSSU),OR139229 (*tef1-α*), and OR139241 (*tub2*)].

Habitat and distribution: sandstone; collected from Sukhothai province, Thailand.

Notes: Some herpotrichiellacious fungi are known to be rock-inhabiting fungi, including *Cladophialophora*, *Exophiala*, *Phialophora*, and *Rhinocladiella* (Coleine and Selbmann, 2021; Liu et al., 2022). *Cladophialophora* is characterized by the production of branched or unbranched chains of one-cell conidia (Badali et al., 2008). *Exophiala* produced annellidic conidiogenesis and yeast-like states (de Hoog et al., 2011). *Phialophora* exhibited the basic morphological characteristics of conidial production through large phialidic conidiogenesis (Li et al., 2017). *Rhinocladiella* and *Petriomyces* share morphological characteristics of being polyblastic, as well as sympodial conidial formation on conidiogenous loci and aseptate conidia (Arzanlou et al., 2007). In addition, the conidia of *Rhinocladiella* appears in various shapes, including subglobose, ellipsoidal, obovoid, and subcylindrical to clavate, and is similar to *Petriomyces*, which appears obovoid or pyriform conidia. However, *Rhinocladiella* commonly produces thick-walled and brown conidiophores (Arzanlou et al., 2007), whereas *Petriomyces* has semi-micronematous, thin-walled, subhyaline, and pale brown conidiophores.

A multi-gene phylogenetic study revealed that *Petriomyces* formed a unique monophyletic clade in *Herprotrichiellaceae* with strong support values (100% BS and 1.00 PP) and formed a sister clade to *Atrokylindriopsis setulose* (HMAS245592) and *Exophiala siamensis* (SDBR-CMU417) (**Figure 1**). *Atrokylindriopsis setulose* was isolated from dead branches of an unidentified broadleaf tree and produced monophialidic conidiogenesis with setulate conidia (Ma et al., 2015). On the other hand, *Petriomyces* was isolated from rocks and produced a sympodial conidial formation with obovoidal conidia. *Petriomyces obovoidisporus* and *Ex. siamensis* were also isolated from rocks. However, *Ex. siamensis* produces short conidiophores, subspherical conidia, and pale brown chlamydospores, whereas *Pe*. *obovoidisporus* produces long conidiophores and obovoidal conidia and does not produce chlamydospores (Thitla et al., 2022). In addition, a pairwise nucleotide comparison of ITS and nrLSU sequencing data between *Petriomyces obovoidisporus* and *Atrokylindriopsis setulose* revealed different values of 12.5% (76/607 bp) and 2.7% (15/548 bp) including gaps. Notably, *Pe. obovoidisporus* and *Ex. siamensis* revealed pairwise nucleotide comparison values of 12.3% (75/609 bp) for ITS, 0.7% (7/1,007 bp) for nrSSU, 26.4% (58/220 bp) for *tef1-α*, and 25.8% (132/512 bp) for *tub2* genes.

#### New species

**Figure 3:**
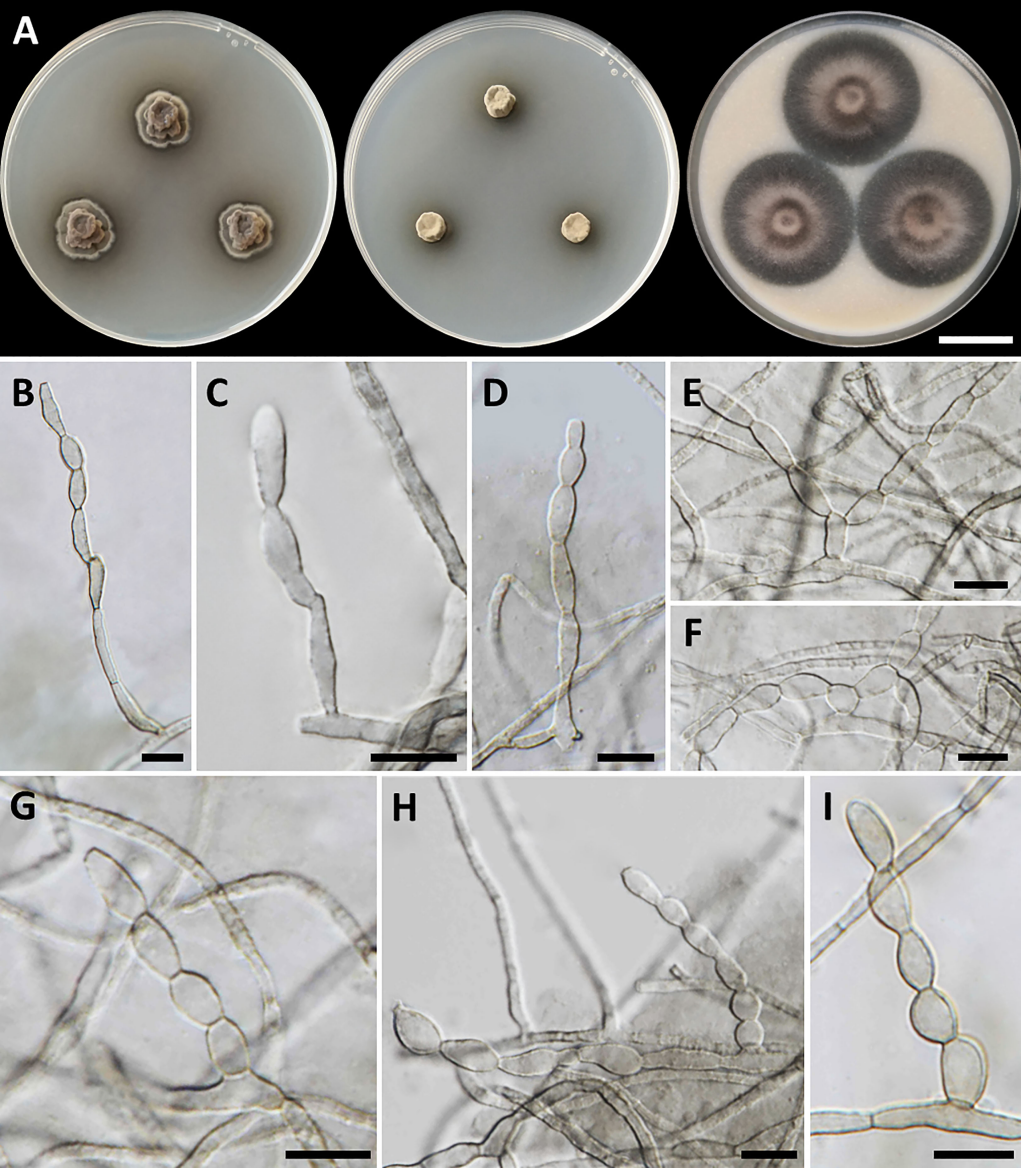
*Cladophialophora rupestricola* (SDBR-CMU446, ex-type). **(A)** Colonies at 25°C for 28 days on PDA, MEA, and OA, respectively. **(B−D)** Conidiophore and conidial chains. **(E−I)** Conidial chains on hyphae. Scale bars: **(A)** 2 cm and **(B−I)** 10 µm.

*Cladophialophora rupestricola*, T. Thitla, J. Kumla, and N. Suwannarach, sp. nov. (**Figure 3**)

MycoBank number: MB846945

Etymology: “*rupestricola*” refers to the rock, where the habitat of the fungus.

Holotype: Thailand, Lamphun province, Mueang Lamphun District, Sribuaban Subdistrict, 18°32′11″N 99°07′22″E, elevation at 444 m, isolated from the sandstone of dipterocarp forest, July 2021, T. Thitla; dried culture, CMULPN5-5; ex-type culture, SDBR-CMU446.

GenBank number: OP903465 (ITS), OP903502 (nrLSU), OR141860 (nrSSU), OP923695 (*tef1-α*), and OR139230 (*tub2*)

Culture characteristics: Colonies on PDA, MEA, and OA were described after 28 days of incubation at 25°C in darkness: on PDA: attaining 15-mm to 17-mm diameter, restricted, circular, convex, velvety, gray in the center with black, and undulate margin; black in reverse; on MEA: attaining 8-mm to 13-mm diameter, restricted, circular, velvety, and dull green with dark green and entire margin; reverse dark green; on OA: attaining 36-mm to 40-mm diameter, restricted, circular, raised centrally, and gray aerial mycelium with greenish gray and entire margin; reverse dark green. A soluble olive and dull green pigment were observed around the fungal colonies on PDA and MEA, respectively. *Hyphae* smooth-walled, septate, hyaline to brown, 1.5-µm to 3-µm wide. *Conidiophores* reduced to conidiogenous cells, lateral or terminal on undifferentiated hyphae, smooth, hyaline to pale olivaceous, cylindrical, and 2.5-µm to 5-µm wide. *Conidia* spherical to ellipsoidal, aseptate, smooth, subhyaline to hyaline, forming branched acropetal chains at hyphae and conidiophore, 6−13.5 µm × 3−6 µm (mean = 9.5 µm × 4.6 µm, *n* = 50); *Chlamydospores* absent. Sexual morph was not produced on media.

Growth temperature: minimum at 15°C, optimum at the range of 25°C−30°C, maximum at 35°C, and no growth at 10°C and 37°C.

Additional specimens examined: Thailand, Lamphun province, Mueang Lamphun District, Sribuaban Subdistrict, 18°32′11″N 99°07′22″E, elevation at 444 m, isolated from the rock of dipterocarp forest, July 2021, isolated by T. Thitla: SDBR-CMU447 [GenBank number: OP903466 (ITS), OP903503 (nrLSU), OR141861 (nrSSU), OP923696 (*tef1-α*), and OR139231 (*tub2*)] and SDBR-CMU448 [GenBank: OP903467 (ITS), OP903504 (nrLSU), OR141862 (nrSSU), OP923697 (*tef1-α*), and OR139232 (*tub2*)].

Habitat and distribution: sandstone; collected from Lamphun province, Thailand.

**Figure 4:**
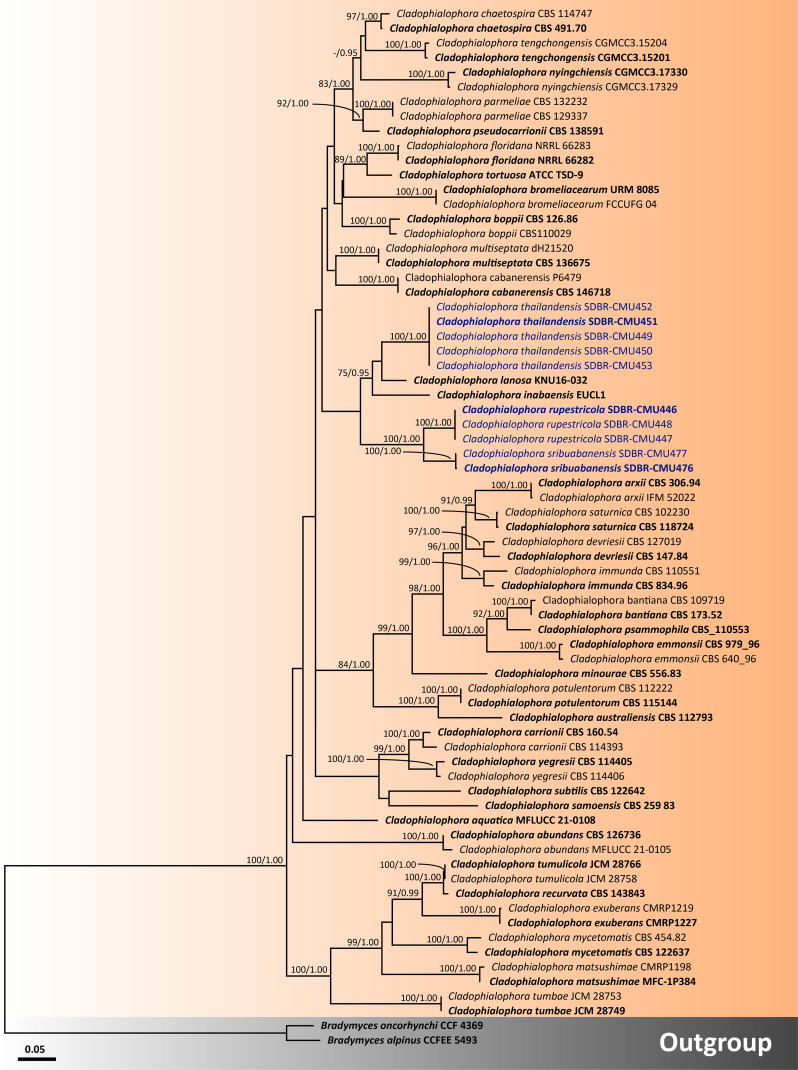
Phylogenetic relationships of *Cladophialophora* within the family *Herpotrichiellaceae* reconstructed by maximum likelihood analysis based on a combined dataset of ITS, nrLSU, nrSSU, *tub2*, and *tef1-α* genes. *Bradymyces alpinus* CCFEE 5493 and *Br. oncorhynchi* CCF 4369 were used as the outgroup. The values presented above branches represent the bootstrap percentages (left) and Bayesian posterior probabilities (right). Bootstrap values ≥75% and Bayesian posterior probabilities ≥0.95 are displayed. The scale bar indicates the estimated number of nucleotide substitutions per site. The newly generated sequences are in blue, whereas the ex-type species are indicated in bold.

Notes: The colony characteristics of *Cl. rupestricola* resembled those of *Cl. chaetospira* and *Cl. floridana* that were isolated from the decaying bamboo and sclerotia of *Cenococcum geophilum*, respectively. However, the conidial size of *Cl. rupestricola* differs from that of *Cl. chaetospira* and *Cl. floridana*. Specifically, the conidia of *Cl. rupestricola* (6−13.5 µm × 3−6 µm) were significantly shorter than that of *Cl. chaetospira* (20−45 µm × 3−5 µm) and slightly broader than that of *Cl. floridana* (3.5−8 µm × 2.0−3µm) (Crous et al., 2007; Obase et al., 2016). In addition, these three species also exhibit distinct conidiophore and conidial characteristics. *Cladophialophora floridana* displayed narrower conidiophores (2-µm to 3-µm wide) than *Cl. repestricola*, which produced broader cylindrical, 2.5-µm- to 5-µm-wide conidiophores (Obase et al., 2016). In terms of conidial characteristics, *Cl. chaetospira* produced 1−3 septate of conidia that is in contrast to *Cl. repestricola*, which produced aseptate conidia (Crous et al., 2007). The multi-gene phylogenetic analyses indicated that *Cl. rupestricola* is a distinct species in *Cladophialophora* and it is closely related to *Cl. sribuabanensis* (**Figure 4**). *Cladophialophora rupestricola* differs from *Cl. sribuabanensis* in terms of the temperature growth profile. *Cladophialophora rupestricola* has a maximum temperature of 35°C, whereas *Cl. sribuabanensis* has a maximum temperature of 30°C. In addition, *Cl. rupestricola* produced a soluble pigment on PDA and MEA, whereas *Cl. sribuabanensis* did not produce any pigment. Furthermore, a pairwise nucleotide comparison of *Cl. rupestricola* and *Cl. sribuabanensis* resulted in differences of 7.0% (45/642 bp) in ITS, 0.2% (2/903 bp) in nrLSU, 0.3% (3/1037 bp) in nrSSU, 9.8% (20/205 bp) in *tef1-α*, and 10.6% (54/509 bp) in *tub2* sequences including gaps.

**Figure 5:**
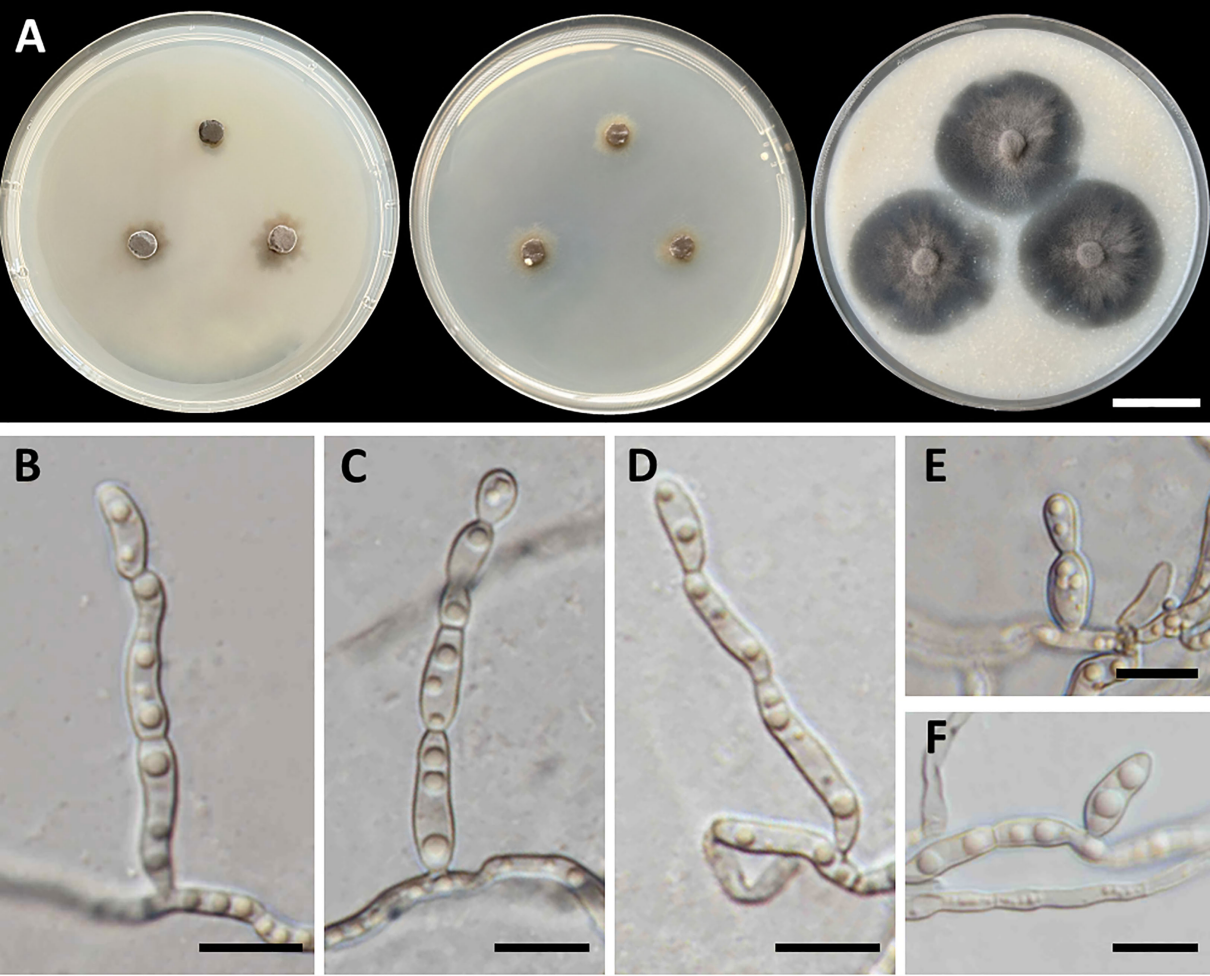
*Cladophialophora sribuabanensis* (SDBR-CMU446, ex-type). **(A)** Colonies at 25°C for 28 days on PDA, MEA, and OA, respectively. **(B–D)** Conidiophore and conidia. **(E−F)** Conidia on hyphae. Scale bars: **(A)** 2–cm and **(B–F)** 10 µm.

*Cladophialophora sribuabanensis*, T. Thitla, N. Suwannarach, and S. Lumyong, sp. nov. (**Figure 5**).

MycoBank number: MB849333

Etymology: “*sribuabanensis*” refers to Sribuaban Subdistrict, Lamphun province, Thailand, where the rock samples were collected.

Holotype: Thailand, Lamphun province, Mueang Lamphun District, Sribuaban Subdistrict, 18°32′11″N 99°07′22″E, elevation at 444 m, isolated from the sandstone of dipterocarp forest, July 2021, T. Thitla; dried culture, CMULPN5-9; ex-type culture, SDBR-CMU476.

GenBank number: OQ991178 (ITS), OQ979608 (nrLSU), OR141868 (nrSSU), OR139226 (*tef1-α)*, and OR139238 (*tub2*)

Culture characteristics: Colonies on PDA and MEA at 25°C extremely slow growing: on PDA: attaining 8-mm to 15-mm diameter; and on MEA: attaining 12-mm to 14-mm diameter in 28 days. The colony character on PDA spreading, circular, flat, olive brown, and undulate margin; brown in reverse; on MEA: circular, spreading, flat, velvety, and grayish green to olive with entire margin; reverse grayish green; on OA: attaining 32-mm to 38-mm diameter, restricted, circular, flat, velvety, and olive brown to olive gray with entire margin; reverse olive gray. No diffusible pigment produced on any media. *Hyphae*: thin and smooth-walled, hyaline to light brown, 2-µm to 3-µm wide. *Conidiophores*: reduced to conidiogenous cells, semi-micronematous, lateral or terminal, smooth, thin-walled, hyaline to pale brown, sub-cylindrical, and 2-µm to 4-µm wide. *Conidia*: subglobose to fusiform, aseptate, guttules, smooth, thin-walled, hyaline to pale brown, forming to unbranched conidial chain at hyphae and conidiophore, and 7−16 µm × 3−6 µm (mean = 11.2 µm × 3.8 µm, *n* = 50). *Chlamydospores* and sexual structures were not produced on any media.

Growth temperature: minimum at 15°C, optimum at the range of 28°C, maximum at 30°C, and no growth at 10°C and 35°C.

Additional specimens examined: Thailand, Lamphun province, Mueang Lamphun District, Sribuaban Subdistrict, 18°32′11″N 99°07′22″E, elevation at 444 m, isolated from the rock of dipterocarp forest, July 2021, isolated by T. Thitla: SDBR-CMU477 [GenBank number: OQ991179 (ITS), OQ979609 (nrLSU), OR141869 (nrSSU), OR139227 (*tef1-α*), and OR139239 (*tub2*)].

Habitat and distribution: sandstone; collected from Lamphun province, Thailand.

Notes: *Cladophialophora sribuabanensis* was isolated from rock samples along with several other species, including *Cl. nyingchiensis*, *Cl. rupestricola*, *Cl. tengchongensis*, *Cl. thailandensis*, *Cl. tumbae*, and *Cl. tumulicola*, all of which were also isolated from rocks (Kiyuna et al., 2018; Sun et al., 2020). However, the conidial size of *Cl. sribuabanensis* (7−16 µm × 3−6 µm) was obviously larger than that of *Cl. tumbae* (4.5−6 µm × 2−2.5 µm) and longer than that of *Cl. tumulicola* (5−7 µm × 3−4 µm). Furthermore, *Cl. nyingchiensis* produced septate conidia, which differs from *Cl*. *sribuabanensis*, which produced aseptate conidia. *Cladophialophora sribuabanensis* showed an exceptionally slow growth rate, reaching only 8−15 mm and 12−14 mm in diameter at 25°C for 28 days on PDA and MEA, respectively. In contrast, both *Cl. tumbae* and *Cl. tumulicola* achieved significantly larger diameters of 15−16 mm and 22−25 mm on PDA, respectively, within just 14 days at 25°C (Kiyuna et al., 2018). *Cladophialophora nyingchiensis*, *Cl. tengchongensis*, and *Cl. thailandensis* also displayed faster growth rates on MEA, attaining diameters of 21 mm, 28 mm, and 23–30 mm, respectively, compared to *Cl*. *sribuabanensis* at the same temperature (Sun et al., 2020). *Cladophialophora sribuabanensis* also differs from *Cl. thailandensis*, which does not produce chlamydospores on any media, whereas *Cl. thailandensis* produces subspherical chlamydospores (7.5−17 µm × 4.5−14 µm). The temperature profile of growth revealed that *Cl. sribuabanensis* has a minimum growth temperature of 15°C, whereas *Cl. nyingchiensis* and *Cl. tengchongensis* could grow at 4°C. In addition, the maximum growth temperature of *Cl. sribuabanensis* is 30°C, whereas *Cl. rupestricola* has a maximum growth temperature of 35°C. Phylogenetically, *Cl. sribuabanensis* forms a sister clade to *Cl. rupestricola* (**Figure 4**). *Cladophialophora sribuabanensis* differs from *Cl. rupestricola* at different maximum growth temperatures (as mentioned above). Moreover, *Cl. sribuabanensis* does not produce any pigments in contrast to *Cl. rupestricola*, which produces olive and dull green pigments on PDA and MEA, respectively. Also, a pairwise nucleotide comparison of *Cl. rupestricola* and *Cl. sribuabanensis* revealed differences of 7.0% (45/642 bp) in the ITS, 0.2% (2/903 bp) in nrLSU, 0.3% (3/1,037 bp) in nrSSU, 9.8% (20/205 bp) in *tef1-α*, and 10.6% (54/509 bp) in *tub2* sequences including gaps.

**Figure 6:**
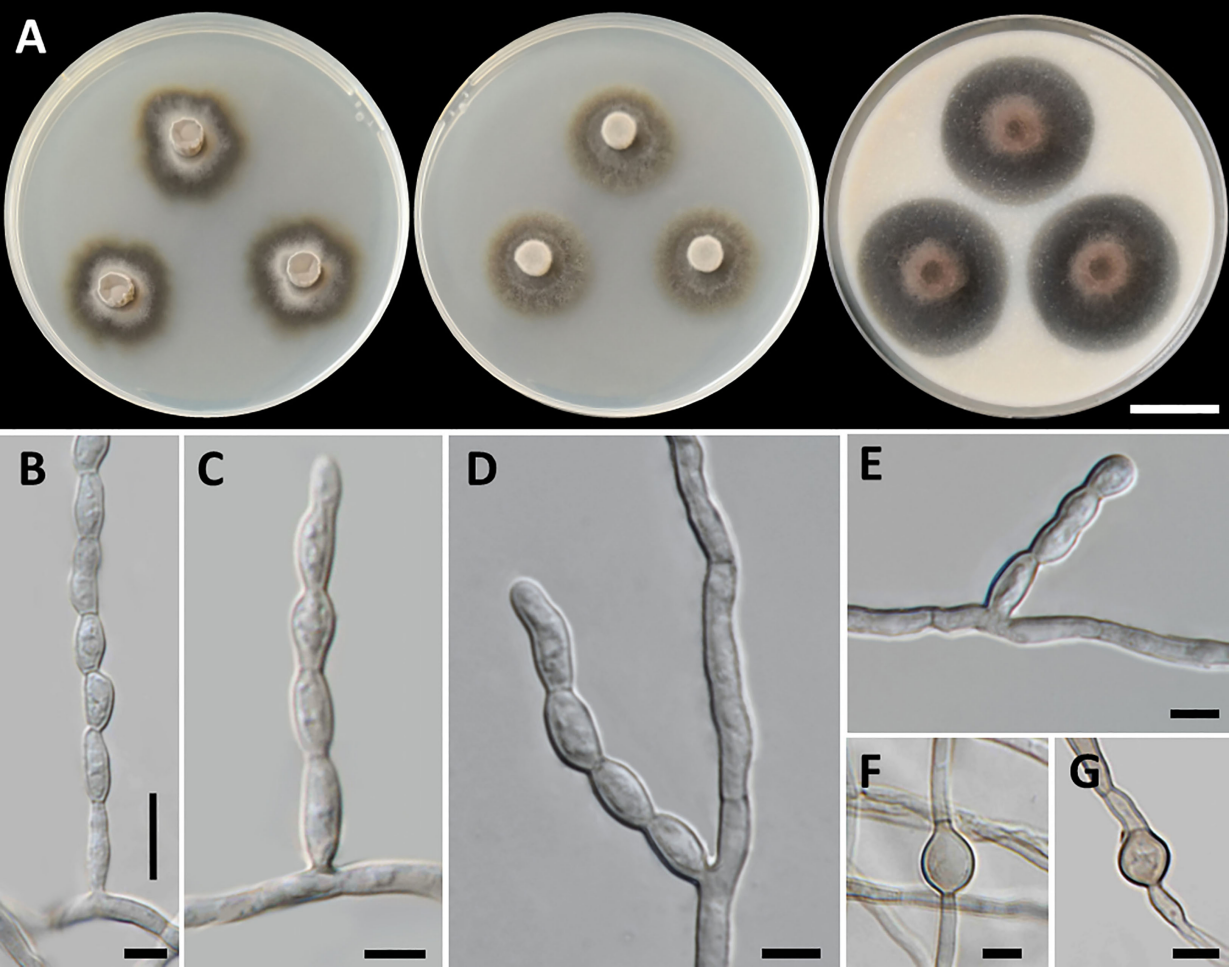
*Cladophialophora thailandensis* (SDBR-CMU451, ex-type), **(A)** Colonies at 25°C for 28 days on PDA, MEA, and OA, respectively. **(B−E)** Conidiophore and conidial chains. **(F, G)** Chlamydospores. Scale bars: **(A)** 2 cm and **(B−G)** 5 µm.

*Cladophialophora thailandensis*, T. Thitla, J. Kumla, and N. Suwannarach, sp. nov. (**Figure 6**)

MycoBank number: MB846946

Etymology: “*thailandensis*” referring to Thailand, where the new species was found.

Holotype: Thailand, Lamphun province, Mueang Lamphun District, Sribuaban Subdistrict, 18°32′13″N 99°07′31″E, elevation at 432 m, isolated from the sandstone of dipterocarp forest, July 2021, T. Thitla; dried culture, CMULPN13-12; ex-type culture, SDBR-CMU451.

GenBank number: OP903470 (ITS), OP903507 (nrLSU), OR141865 (nrSSU), OP923700 (*tef1-α*), and OR139235 (*tub2*).

Culture characteristics: Colonies on PDA, MEA, and OA were described at 25°C in the dark after 28 days: on PDA: attaining 28-mm to 34-mm diameter, irregularly circular, flat, center velvety gray to black with grayish green and entire to undulate margin; black to grayish green in reverse; on MEA: attaining 23-mm to 30-mm diameter, spreading, circular, velvety, and greenish gray to dark green with grayish green and entire margin; reverse black to grayish green; on OA: attaining 32-mm to 39-mm diameter, restricted, circular, convex, and olive brown aerial mycelium with dark green and entire margin; reverse dark green to dull green. No diffusible pigment produced on any media. *Hyphae* smooth-walled, septate, hyaline to pale brown, 1.5-µm to 4-µm wide. *Conidiophores*: reduced to conidiogenous cells, lateral on undifferentiated hyphae, producing long chains conidia, smooth, pale olivaceous, cylindrical to subcylindrical, and 2-µm to 4-µm wide. *Conidia*: subspherical to fusiform, aseptate, subhyaline to hyaline, smooth, acropetal, catenulate, and 5−11 µm × 3−4 µm (mean = 7.3 µm × 3.3 µm, *n* = 50). *Chlamydospores*: subspherical, smooth, light to dark brown, and 7.5−17 µm × 4.5−14 µm (mean = 10.7 µm × 7.2 µm, *n* = 20). Sexual morph was not produced on media.

Growth temperature: minimum at 10°C, optimum at 28°C, maximum at 35°C, and no growth at 4°C and 37°C.

Additional specimens examined: Thailand, Lamphun province, Mueang Lamphun District, Sribuaban Subdistrict, 18°32′13″N 99°07′31″E, elevation at 432 m, isolated from the rock of dipterocarp forest, July 2021, isolated by T. Thitla SDBR-CMU449 [GenBank number: OP903468 (ITS), OP903505 (nrLSU), OR141863 (nrSSU), OP923698 (*tef1-α*), and OR139233 (*tub2*)], SDBR-CMU450 [GenBank number: OP903469 (ITS), OP903506 (nrLSU), OR141864 (nrSSU), OP923699 (*tef1-α*), and OR139234 (*tub2*)], and SDBR-CMU452 [GenBank number: OP903471 (ITS), OP903508 (nrLSU), OR141866 (nrSSU), OP923701 (*tef1-α*), and OR139236 (*tub2*)]; rock from dipterocarp forest, August 2022, isolated by T. Thitla SDBR-CMU453 [GenBank number: OP903472 (ITS), OP903509 (nrLSU), OR141867 (nrSSU), OP923702 (*tef1-α*), and OR139237 (*tub2*)].

Habitat and distribution: sandstone; known from Lamphun province, Thailand.

Notes: *Cladophialophora thailandensis* produced a greenish gray colony on MEA. This outcome was similar to that of *Cl. eucalypti*, *Cl. immunda*, *Cl. mycetomatis*, and *Cl. pseudocarrionii*. Nevertheless, *Cl*. *thailandensis* has distinct habitats from these species. *Cladophialophora eucalypti* was isolated from leaves of *Eucalyptus dunnii*, whereas *Cl*. *pseudocarrionii* originated from soil, and both *Cl*. *immunda* and *Cl*. *mycetomatis* were isolated from humans (Badali et al., 2008; Madrid et al., 2016; Crous et al., 2019a). Furthermore, the conidial size of *Cl. thailandensis* (5−11 µm × 3−4 µm) is shorter than that of *Cl. eucalypti* (8−20 µm × 2.5−3 µm) (Crous et al., 2019a) but longer than that of *Cl. immunda* (3−4.5 µm × 2.5−4 µm) and *Cl. mycetomatis* (2.5−3 µm × 2−3 µm) (Badali et al., 2008). *Cladophialophora thailandensis* can be distinguished from *Cl. pseudocarrionii* by having a subspherical chlamydospore, whereas the chlamydospore production of *Cl. pseudocarrionii* was not observed (Madrid et al., 2016). In terms of the growth temperature, *Cl. thailandensis* also differs from *Cl. immunda*, *Cl. mycetomatis*, and *Cl. pseudocarrionii*, as *Cl. thailandensis* is unable to grow at 37°C (Badali et al., 2008; Madrid et al., 2016).

The multi-gene phylogenetic analyses indicated that *Cl. thailandensis* formed a distinct monophyletic clade within *Cladophialophora* and was closely related to *Cl. inabaensis* and *Cl. lanosa* isolated from soil samples (**Figure 4**). However, the absence of chlamydospore formation in *Cl. inabaensis* is differentiated from *Cl. thailandensis* (Usui et al., 2016). In contrast to *Cl*. *thailandensis*, which exhibited a maximum growth temperature of 35°C, *Cl. inabaensis* could grow at 37°C, indicating a higher tolerance to elevated temperatures (Usui et al., 2016). Moreover, the conidia of *Cl. thailandensis* are longer than those of *Cl. lanosa* (2−4 µm × 2−3 µm) (Das et al., 2019). A pairwise nucleotide comparison between the ITS sequence data of *Cl. thailandensis* to *Cl. inabaensis* and *Cl. lanosa* indicated base pair differences of 8.8% (45/510 bp) and 6.9% (36/515 bp), respectively. In addition, a pairwise nucleotide comparison of nrLSU data also indicated that *Cl. thailandensis* differs from *Cl. lanosa* by 0.5% (4/764 bp) including gaps.

## Discussion

*Herpotrichiellaceae* is one of the well-known families in the order *Chaetothyriales* (Wijayawardene et al., 2020; Tian et al., 2021; Bezerra et al., 2022; Wijayawardene et al., 2022). Presently, four genera in *Herpotrichiellaceae* have been discovered on rocks, *viz.*, *Cladophialophora*, *Exophiala*, *Phialophora*, and *Rhinocladiella* (Sun et al., 2020; Liu et al., 2022; Thitla et al., 2022). In this study, we introduced new taxa of rock-inhabiting fungi in the family *Herpotrichiellaceae* that were collected from northern Thailand. These taxa comprised a novel genus, named *Petriomyces* and three new species of *Cladophialophora*. The genus *Petriomyces* has been introduced on the basis of its fungal habitat, morphological characteristics, growth temperature, and phylogenetic analysis. Consequently, the number of rock-inhabiting fungal genera in the family *Herpotrichiellaceae* has increased to five genera.

Prior to this study, 20 genera (*Aculeata*, *Atrokylindriopsis*, *Brycekendrickomyces*, *Capronia*, *Cladophialophora*, *Exophiala*, *Fonsecaea*, *Marinophialophora*, *Melanoctona*, *Metulocladosporiella*, *Minimelanolocus*, *Neosorocybe*, *Phialophora*, *Pleomelogramma*, *Rhinocladiella*, *Sorocybe*, *Thysanorea*, *Uncispora*, *Valentiella*, and *Veronaea*) have been accepted into the family *Herpotrichiellaceae* (Wijayawardene et al., 2020; Tian et al., 2021; Bezerra et al., 2022; Wijayawardene et al., 2022). However, the number of genera in *Herpotrichiellaceae* has been a subject of debate in previous studies due to the variability of their morphological characteristics (Quan et al., 2020; Tian et al., 2021). Therefore, molecular phylogeny has been recognized as a valuable tool for researchers in the identification of fungi within *Herpotrichiellaceae*. Consequently, the aim of this study was to inspect and update the members of this family through molecular phylogeny using combined ITS, nrLSU, and nrSSU sequences. In our phylogenetic tree (**Figure 1**) indicated that species of herpotrichiellaceous fungi are paraphyletic and polyphyletic within the family *Herpotrichiellaceae* and *Trichomeriaceae* and this is concurred with previous studies (Quan et al., 2020; Wan et al., 2021).

A new genus (*Petriomyces*) obtained from this study formed within *Herpotrichiellaceae* (**Figure 1**). Regarding species *Brycekendrickomyces* and *Metulocladosporiella*, which were previously placed in the family *Herpotrichiellaceae* (Crous et al., 2006; Crous et al., 2009; Wijayawardene et al., 2020; Tian et al., 2021; Bezerra et al., 2022; Wijayawardene et al., 2022), our phylogenetic analysis placed their type sequences within the family *Trichomeriaceae*. Morphologically, these two species were reported from asexual characters, whereas the members of family *Trichomeriaceae* are predominantly known for their sexual characters. Although, there have been reports of asexual reproduction in certain species within family *Trichomeriaceae*, the specific asexual characters vary within the family. Because of the variability of asexual characters within the family *Trichomeriaceae*, we have tentatively transferred genera *Brycekendrickomyces* and *Metulocladosporiella* to the family *Trichomeriaceae* based solely on their phylogenetic placement. However, further studies, including additional collections and the linkage of sexual and asexual morphs for these two species, are necessary to confirm their phylogenetic placement and to gain a better understanding of the diversity of asexual characters within the family *Trichomeriaceae*. Furthermore, *Neosorocybe* and *Sorocybe* were also previously classified into *Herpotrichiellaceae* (Crous et al., 2020b; Wijayawardene et al., 2022). However, the phylogenetic analysis in this study revealed that these genera formed a unique clade to be clearly distinct from *Herpotrichiellaceae* and *Trichomeriaceae* (**Figure 1**). These two genera should be excluded from the family *Herpotrichiellaceae*. Therefore, we propose that the family *Herpotrichiellaceae* should contain 17 fungal genera (**Table 4**). However, there is still a need for the molecular data of the genus *Pleomelogramma* (Spegazzini, 1909) to confirm its phylogenetic placement. Most herpotrichiellaceous fungi still have a broadly paraphyletic and polyphyletic relationship. Therefore, further resolutions and characterizations are required to achieve a better understanding of their taxonomic classification within *Herpotrichiellaceae*.

**Table 4:** Comparison of fungal genera belonging to the family *Herpotrichiellaceae* from previous reports and this study. .

Fungal Genus	Fungal Family*				
	Wijayawardene et al. (2020)	Tian et al. (2021)	Wijayawardene et al. (2022)	Bezerra et al. (2022)	This study (2023)
*Aculeata*	◼	◼	◼	◼	◼
*Atrokylindriopsis*	▴	◼	▴	◼	◼
*Brycekendrickomyces*	◼	◼	◼	◼	●
*Capronia*	◼	◼	◼	◼	◼
*Cladophialophora*	◼	◼	◼	◼	◼
*Exophiala*	◼	◼	◼	◼	◼
*Fonsecaea*	◼	◼	◼	◼	◼
*Marinophialophora*	◼	◼	◼	◼	◼
*Melanoctona*	◼	◼	◼	◼	◼
*Metulocladosporiella*	◼	◼	◼	◼	●
*Minimelanolocus*	◼	◼	◼	◼	◼
*Neosorocybe*	NR	NR	◼	NR	▴
*Petriomyces*	NR	NR	NR	NR	◼
*Phialophora*	◼	◼	◼	◼	◼
*Pleomelogramma*	◼	◼	◼	◼	◼
*Rhinocladiella*	◼	◼	◼	◼	◼
*Sorocybe*	◼	◼	◼	◼	▴
*Thysanorea*	◼	◼	◼	◼	◼
*Uncispora*	▴	◼	▴	◼	◼
*Valentiella*	NR	NR	NR	◼	◼
*Veronaea*	◼	◼	◼	◼	◼

The fungal family is represented by a color symbol; *Herpotrichiellaceae* is shown by ◼, *Trichomeriaceae* is indicated by ●, incertae sedis in class *Chaetothyriales* is indicated by ▴, and not reported is indicated by “NR.”

In this study, the phylogenetic results indicate that *Cladophialophora* is a polyphyletic group, as is consistent with the findings of previous studies that reported the polyphyletic nature of the *Cladophialophora* species (Badali et al., 2008; Teixeira et al., 2017; Quan et al., 2020). Our three new species of *Cladophialophora* (*Cl*. *rupestricola*, *Cl*. *sribuabanensis*, and *Cl*. *thailandensis*) were established on the basis of comprehensive analyses of morphological characteristics, growth temperature, and multi-gene phylogeny. Furthermore, the nucleotide comparisons of the ITS, *tef1-α*, or *tub2* genes with phylogenetically related species revealed nucleotide differences that were greater than 1.5%. This is in line with the suggestion made by Jeewon and Hyde (2016) that nucleotide differences above 1.5% are necessary to justify the recognition of a new species. Therefore, *Cl*. *rupestricola*, *Cl*. *sribuabanensis*, and *Cl*. *thailandensis* can be considered new species, whereas the total number of accepted *Cladophialophora* species globally has increased to 52. On the basis of our phylogenetic analysis, a total of 38 *Cladophialophora* species were classified as members of *Herpotrichiellaceae*. Three species (*Cl*. *eucalypti*, *Cl*. *proteae*, and *Cl*. *pucciniophila*) were placed in *Trichomeriaceae*, which is consistent with the study of Quan et al. (2020). Moreover, Quan et al. (2020) reported that *Cl*. *humicola*, *Cl*. *minutissima*, and *Cl*. *sylvestris* were placed in *Epibryaceae*. Accordingly, *Cl. behniae*, *Cl. modesta*, *Cl. scillae*, and *Cl. hostae* have been assigned in an incertae sedis clade *Chaetothyriales* (Quan et al., 2020; Crous et al., 2021). However, the phylogenetic placements of the remaining four species (*Cl. cladoniae*, *Cl. hawksworthii*, *Cl. megalosporae*, and *Cl. normandinae*) are uncertain and the acquisition of further molecular data will be necessary.

An investigation of fungal grown at variant temperatures indicates that our fungi could grow under a wide range of temperature conditions. Three new species of *Cladophialophora* could thrive at temperatures ranging from 10°C to 35°C. Moreover, a new genus *Pe. obovoidisporus* could thrive at temperatures ranging between 10°C and 30°C. Accordingly, these temperatures were within a range of 10°C−38°C in the dipterocarp forest in Lamphun and Sukhothai provinces, Thailand, respectively. As has become evident, both fungi have adapted to survive in their respective environments. Remarkably, growth temperature is one of the factors that can limit fungal ability to infect human and animal bodies due to their high body temperatures (Garcia-Solache and Casadevall, 2010). Some herpotrichiellaceous fungi that are known to infect humans and animals can grow at high temperatures. For example, *Cl. arxii*, *Cl. carrionii*, *Phia*. *chinensis*, and *Phia*. *expanda* can grow at temperatures of 37°C or higher (de Hoog et al., 1995; Li et al., 2017). The growth temperature of herpotrichiellaceous fungi is often associated with clinical predilections with the species growing at 40°C often causing systemic infections (de Hoog et al., 2000; Badali et al., 2008). Three new species of *Cladophialophora* and *Petriomyces* identified in this study may not be the source pathogen in humans and animals because they cannot grow at temperatures above 37°C. However, their ability to be the causal agents of infections in humans and animals needs to be further investigated. Currently, global temperatures are rising because of climate change, which can affect all aspects of a given ecosystem (Gadre et al., 2022). As a result, this environment may have an impact on the global diversity and distribution of herpotrichiellaceous fungi. Some herpotrichiellace fungi can survive and grow at high temperatures, but others cannot survive at higher temperatures and die.

In conclusion, we investigated the rock-inhabiting fungi belonged to the family *Herpotrichiellaceae* collected from northern Thailand. Three new species of *Cladophialophora* (*Cl*. *rupestricola*, *Cl*. *sribuabanensis*, and *Cl*. *thailandensis*) and a new genus (*Petriomyces*) were identified on the basis of the relevant morphological characteristics, growth temperature, and multi-gene phylogeny. Furthermore, the relevant data on the genera in *Herpotrichiellaceae* have been updated. In this study, we propose that *Herpotrichiellaceae* consists of 17 genera, including *Aculeata*, *Atrokylindriopsis*, *Capronia*, *Cladophialophora*, *Exophiala*, *Fonsecaea*, *Marinophialophora*, *Melanoctona*, *Minimelanolocus*, *Petriomyces*, *Phialophora*, *Pleomelogramma*, *Rhinocladiella*, *Thysanorea*, *Uncispora*, *Valentiella*, and *Veronaea*. However, the classification of several genera in this family is still problematic due to variabilities in some of the morphological characteristics, their existing polyphyletic relationships, and the limited amount of molecular data, especially protein coding genes. Therefore, further studies involving morphology, ecology, and molecular investigations, as well as in-depth evolutionary studies, should be conducted to further clarify the family *Herpotrichiellaceae*.

## Data availability statement

The datasets presented in this study can be found in online repositories. The names of the repository/repositories and accession number(s) can be found in the article/supplementary material.

## Author contributions

NS and SL: conceptualization in this study. TT, JK, and NS: methodology. TT, JK, SH, CS, and NS: software. TT, JK, and NS: validation. TT, SK, and JK: formal analysis. TT, JK, and NS: investigation. TT, SK, JK, and NS: resources. TT and JK: data curation. TT, JK, and NS: writing—original draft preparation. TT, JK, SH, CS, SK, SL, and NS: writing—review and editing. JK, SH, and NS: visualization. SL and NS: supervision. NS and JK: project administration. SL and NS: funding acquisition. All authors have read and agreed to the published version of the manuscript.
